# Genomic Signatures for Species-Specific Adaptation in Lake Victoria Cichlids Derived from Large-Scale Standing Genetic Variation

**DOI:** 10.1093/molbev/msab084

**Published:** 2021-03-21

**Authors:** Haruna Nakamura, Mitsuto Aibara, Rei Kajitani, Hillary D J Mrosso, Semvua I Mzighani, Atsushi Toyoda, Takehiko Itoh, Norihiro Okada, Masato Nikaido

**Affiliations:** 1 School of Life Science and Technology, Tokyo Institute of Technology, Tokyo, Japan; 2 Tanzania Fisheries Research Institute (TAFIRI), Mwanza Fisheries Research Center, Mwanza, Tanzania; 3 Tanzania Fisheries Research Institute (TAFIRI), Headquarters, Dar es Salaam, Tanzania; 4 Fisheries Education and Training Agency, Dar es Salaam, Tanzania; 5 Comparative Genomics Laboratory, National Institute of Genetics, Mishima, Shizuoka, Japan; 6 School of Pharmacy, Kitasato University, Kanagawa, Japan

**Keywords:** Lake Victoria, cichlids, adaptation, comparative genome, standing genetic variation

## Abstract

The cichlids of Lake Victoria are a textbook example of adaptive radiation, as >500 endemic species arose in just 14,600 years. The degree of genetic differentiation among species is very low due to the short period of time after the radiation, which allows us to ascertain highly differentiated genes that are strong candidates for driving speciation and adaptation. Previous studies have revealed the critical contribution of vision to speciation by showing the existence of highly differentiated alleles in the visual opsin gene among species with different habitat depths. In contrast, the processes of species-specific adaptation to different ecological backgrounds remain to be investigated. Here, we used genome-wide comparative analyses of three species of Lake Victoria cichlids that inhabit different environments—*Haplochromis chilotes*, *H. sauvagei*, and *Lithochromis rufus*—to elucidate the processes of adaptation by estimating population history and by searching for candidate genes that contribute to adaptation. The patterns of changes in population size were quite distinct among the species according to their habitats. We identified many novel adaptive candidate genes, some of which had surprisingly long divergent haplotypes between species, thus showing the footprint of selective sweep events. Molecular phylogenetic analyses revealed that a large fraction of the allelic diversity among Lake Victoria cichlids was derived from standing genetic variation that originated before the adaptive radiation. Our analyses uncovered the processes of species-specific adaptation of Lake Victoria cichlids and the complexity of the genomic substrate that facilitated this adaptation.

## Introduction

Understanding the evolutionary processes of organisms at the molecular level is an important issue in evolutionary biology. The cichlids of three East African Great Lakes, Lake Tanganyika, Malawi, and Victoria are highly diverse in their ecology and morphology. As the great diversity of cichlids is believed to have originated from a small number of ancestral species through adaptive radiation, they are frequently studied examples of speciation and adaptation (Brawand et al. 2014; Salzburger 2018). In particular, Lake Victoria contains >500 endemic species that were generated in just 14,600 years after it dried up at the end of the Pleistocene (Johnson et al. 2000). The level of genetic differentiation among species is considered to be very low due to the short period of time after speciation (Samonte et al. 2007), which gives us a great opportunity to find candidate genes that have contributed to speciation and adaptation. In other words, the genetic differentiation among species can be used to find the signatures of natural selection that occurred during the speciation and adaptation of Lake Victoria cichlids. A prominent example of one such process is the long-wavelength-sensitive opsin gene (*LWS*). Divergent alleles of *LWS* are variable among species in Lake Victoria depending on their different light environments, such as habitat depths and turbidities; this variability has led to rapid ecological speciation via sensory drive (Terai et al. 2002; Seehausen et al. 2008; Miyagi et al. 2012). Also, *V1R2*, which encodes a pheromone receptor, has two main divergent alleles among species in Lake Victoria (Nikaido et al. 2014). Thus, the allelic diversity of genes related to sensory perception may reflect the contribution of mating preference in cichlids to speciation.

The presence of differentiated genomic regions among cichlids regardless of whether they are coding or noncoding regions is an important factor for understanding speciation and adaptation processes. They also allow us to ask broader questions, such as how cichlids have acquired the genomic substrate that enabled the rapid adaptation to local environments and what genetic mechanisms have been involved in this process. One possible explanation could be positive selection against standing genetic variation (SGV), which is retained at a certain frequency within a population and enables beneficial alleles to spread and fix within the population rapidly when environmental changes occur (Barrett and Schluter 2008; Hermisson and Pennings 2017; Marques et al. 2019). SGV is acquired by the retention of the ancestral polymorphisms or hybridization. For example, the genetic polymorphism of endemic Lake Malawi cichlids acquired by hybridization between lake and riverine species was broadly shared with other East African cichlids, which could facilitate the radiation (Loh et al. 2013; Malinsky et al. 2018; Svardal et al. 2020). In Lake Victoria, some differentiated single-nucleotide polymorphisms (SNPs) among cichlid species are shared with the cichlids of other lakes (Brawand et al. 2014). The highly divergent alleles of *V1R2* in Lake Victoria cichlids originated before the radiation of the cichlids of the three East African Great Lakes (Nikaido et al. 2014). The differentiated SNPs among Lake Victoria cichlids were obtained by hybridization between two parental lineages before adaptive radiation (Meier et al. 2017). Most recent evolutionary biological study underscored the importance of genomic potential on the explosive adaptation of Lake Victoria cichlids by showing the ancient haplotypes with insertion/deletion polymorphisms shared with other East African cichlids (McGee et al. 2020). Thus, the exploration of the divergent alleles whose origins are older than the radiation of Lake Victoria cichlids is of primary interest because they may play an important role in rapid adaptation.

Recently, genome-wide comparative analyses have been used to explore highly differentiated genes among species that contribute to speciation and adaptation (Brawand et al. 2014; Salzburger 2018). For example, Meier et al. (2018) found that many candidate genes related to vision, morphological development and behavior contributed to incipient adaptation according to habitat depth based on whole-genome comparative analyses of two Lake Victoria cichlid species that inhabit rocky areas, *Pundamilia nyererei*, which is found in deep waters, and *Pundamilia pundamilia*, which is found in shallow waters. Also, Takuno et al. (2019) examined the patterns of genetic differentiation between two Lake Victoria cichlid species that inhabit different depths on a sandy-muddy bottom and revealed that the two species showed remarkable differences in the expression of candidate genes with differentially fixed SNPs. The aforementioned studies on Lake Victoria cichlids mainly focused on the speciation mechanism of closely related species pairs inhabiting different depths and thus different light environments. However, the processes of adaptation of cichlids with different ecological backgrounds, such as lake bottom environments remain to be investigated in terms of the population history of these species and candidate genes. In addition, until now, a comprehensive analysis focusing on the extent and age of SGV in cichlid genomes has been lacking.

In this study, we focused on three Lake Victoria cichlid species, *Haplochromis chilotes*, *H. sauvagei*, and *Lithochromis rufus*, which inhabit different lake bottom environments, to examine species-specific adaptation processes. Note that we particularly address the elucidation of the adaptation process of each species by including data from *H. sauvagei*, which diverged earlier than the other species of Lake Victoria cichlids (Samonte et al. 2007; Takeda et al. 2013). First, we estimated the population structure of the three species and inferred the pattern of changes in the population size of each species. Then, we explored the genes that are located in highly differentiated regions (HDRs) between species by focusing on two criteria, *F*_ST_ and *d*_XY_, the measures of population differentiation. Finally, we explored the origins of these divergent alleles by molecular phylogenetic analyses using nine species including cichlids from other lakes and rivers. Our comprehensive population genetics analyses revealed the distinct patterns of population history among species according to habitats. We also revealed a substantial number of highly differentiated alleles in Lake Victoria cichlids derived from SGV that originated before the adaptive radiation. The results of the present study provide additional insights into the tempo, mode and mechanism of adaptive radiation of East African cichlids.

## Results

### Statistical Values

**Fig. 1.**
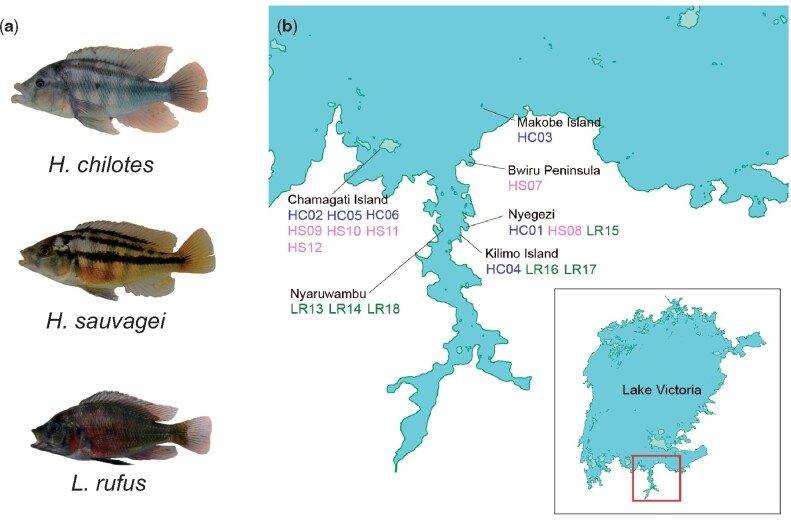
(*a*) Photos of *Haplochromis chilotes*, *H. sauvagei*, and *Lithochromis rufus*. (*b*) Sampling points in Mwanza Gulf and its surroundings in Lake Victoria. Each color and two-letter code indicate the different species: *H. chilotes* (blue, HC), *H. sauvagei* (pink, HS), and *L. rufus* (green, LR), and each number corresponds to the individual name as listed in supplementary table S1, Supplementary Material online.

We used whole-genome sequencing data from six individuals each of three cichlid species, *H. chilotes*, *H. sauvagei*, and *L. rufus* from Mwanza Gulf and its surroundings in Lake Victoria (fig. 1*a* and *b* and supplementary table S1, Supplementary Material online). *Haplochromis chilotes* and *H. sauvagei* inhabit only rocky shores (rocky specialists), and *L. rufus* inhabits various bottom environments (generalist), such as rocky and sandy areas and areas with vegetation. Individuals of each species were collected from several sampling points. After mapping and variant calling against the de novo-assembled genome of *H. chilotes* and extracting sites without insertions and deletions under our criteria (see Materials and Methods), we obtained 1,879,895 biallelic SNPs. The mean of nucleotide diversity per site (*π*) within species was 0.000501 in *H. chilotes*, 0.000403 in *H. sauvagei*, and 0.000563 in *L. rufus.* Based on a calculated inbreeding coefficient for each individual, the values of all individuals of *L. rufus* were negative (supplementary fig. S1, Supplementary Material online). We also calculated the genome-wide *F*_ST_ (Weir and Cockerham 1984) to evaluate the genetic distance between each pair of species. Its weighted mean was 0.21848 between *H. sauvagei* and *H. chilotes*, 0.16926 between *H. sauvagei* and *L. rufus*, and 0.090604 between *H. chilotes* and *L. rufus*.

### Population History

**Fig. 2.**
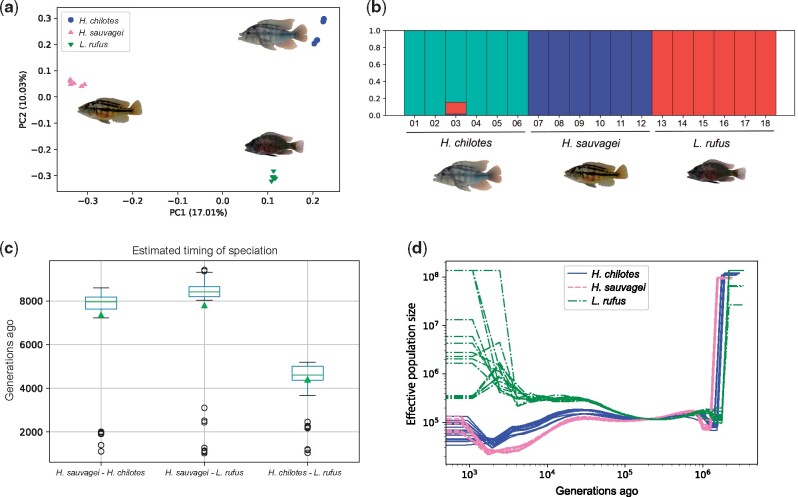
For the following two analyses, 61,461 LD-pruned SNPs depicted the genetic structure for each individual in our study. (*a*) PC1 and PC2 obtained by a PCA based on SNPs for all individuals of *Haplochromis chilotes*, *H. sauvagei*, and *Lithochromis rufus*. (*b*) Results of ADMIXTURE analyses of all individuals with *K *=* *3. The genetic structure of each individual is shown; the numbering along the *x* axis corresponds to the individual names in supplementary table S1, Supplementary Material online. (*c*) The timings of speciation between pairs of species estimated by *smc++* analyses with 100 bootstrap replicates. The average values of the timings of speciation between pairs of species are shown by green triangles. All results are estimated by using 12 unphased haplotypes (six individuals of each species) data comprised from SNPs on the same scaffolds as msmc2 analyses. We used 3.5 × 10^−9^ mutations per base pair per generation as the mutation rate (Malinsky et al. 2018). (*d*) The pattern of changes in population size over generations for *H. chilotes*, *H. sauvagei*, and *L. rufus* as inferred by *msmc2* with genome-wide data from four haplotypes per species. We used a mutation rate of 3.5 × 10^−9^ mutations per base pair per generation (Malinsky et al. 2018).

Differences in ecological backgrounds of species could affect population history. Takeda et al. (2013) showed that the population structure among cichlid species of Lake Victoria was mainly separated by habitats. In addition, they indicated a recent population expansion of Lake Victoria cichlids using the values of neutrality statistics. Given that the population size affects the rate of gene substitution under nearly neutral theory (Ohta 1972), it is important to determine the population history to elucidate the processes of adaptation and speciation of cichlids. To examine whether or not genetic exchanges occurred among the three species, we performed principal component analysis (PCA) against SNP data. Here, we used 61,461 SNPs after removing SNPs with minor allele frequency <0.05 and pruning those by the degree of linkage disequilibrium. In both PC1 and PC2, all individuals were divided into three genetic clusters that reflect each species (fig. 2*a*). In PC3, *H. chilotes* separated into genetic clusters that reflect sampling points, indicating that *H. chilotes* had several local subpopulations (supplementary fig. S2, Supplementary Material online). ADMIXTURE analyses produced a similar result in the case of *K *=* *3 (fig. 2*b*, supplementary fig. S3, Supplementary Material online). In these analyses, the validation error rate was lower for *K *=* *2 than *K *=* *3 (supplementary fig. S4, Supplementary Material online). We suggest that this resulted from a low degree of genetic differentiation among Lake Victoria cichlids.

We estimated the timing of speciation between pairs of species using the *smc++* program for all individuals of each species with 100 bootstrap replicates (fig. 2*c*). The average timing of speciation between *H. chilotes* and *L. rufus* was approximately 8,800 years ago assuming a generation time of 2 years, which was the most recent in the pairwise comparisons of these three species. The speciation was estimated to occur approximately 14,700 years ago between *H. sauvagei* and *H. chilotes* and 15,600 years ago between *H. sauvagei* and *L. rufus*. From these analyses, we propose that *H. sauvagei* diverged earlier than the other two cichlids, which is consistent with our values for *F*_ST_ and with findings from previous studies (Samonte et al. 2007; Takeda et al. 2013).

To examine the pattern of changes in the population size of each species, we ran the *msmc2* program (Schiffels and Durbin 2014; Malaspinas et al. 2016) with 383 scaffolds, each of which was longer than 500 kb, for pairs of individuals in each species. The results showed that these patterns differed substantially according to habitats (fig. 2*d*). Two rocky-specialist species, *H. chilotes* and *H. sauvagei*, experienced a reduction in population size approximately 20,000 years ago and recovered approximately 2,000 years ago (again, with a generation time of 2 years). The degree of the reduction of *H. sauvagei* was larger than that of *H. chilotes*. By contrast, *L. rufus* maintained a larger population size as compared with the other two species.

### Highly Differentiated Regions

**Fig. 3.**
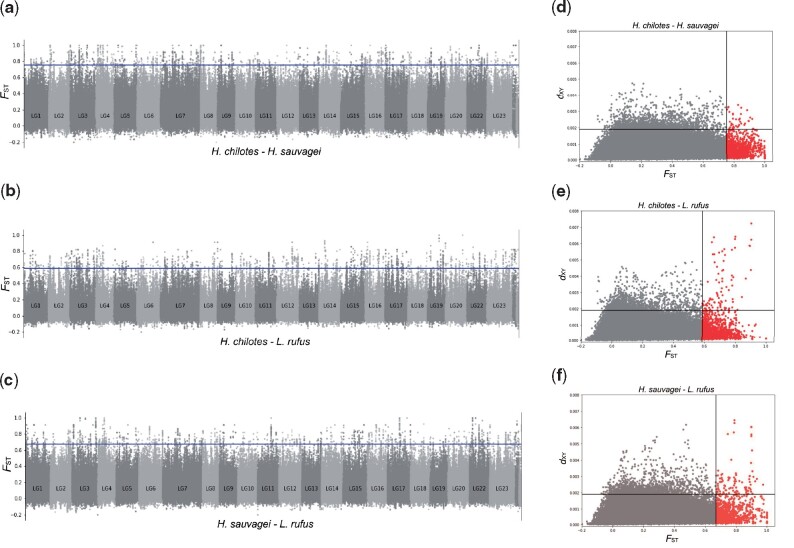
Genome-wide pattern of genetic differences between species pairs. (*a*–*c*) Genome-wide *F*_ST_ values for each linkage group. All values were calculated in overlapping windows of 10 kb. The blue line indicates the top 0.5% of values. Species pairs used for the calculations are (*a*) *Haplochromis chilotes* and *H. sauvagei*, (*b*) *H. chilotes* and *Lithochromis rufus* and (*c*) *H. sauvagei* and *L. rufus*. (*d*–*f*) Each plot explains the genetic difference in a certain window of 10 kb by calculating the weighted mean of *F*_ST_ and *d*_XY_. Red plots show highly differentiated regions between the pair of species. The two black solid lines represent the top 0.5% of values for each statistic. Species pairs used for calculation are (*d*) *H. chilotes* and *H. sauvagei*, (*e*) *H. chilotes* and *L. rufus*, and (*f*) *H. sauvagei* and *L. rufus*.

**Fig. 4.**
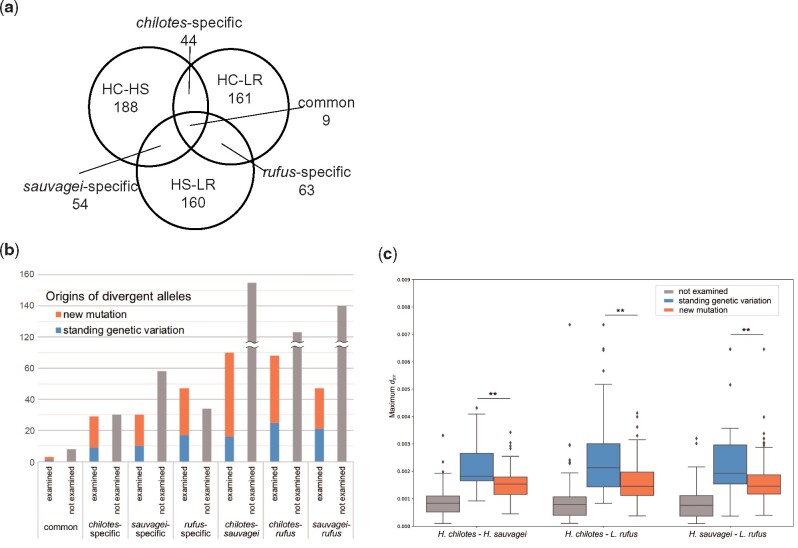
(*a*) Venn diagram showing the number of genes located on HDRs. (*b*) Number of genes with a divergent allele that originated before the adaptive radiation of Lake Victoria cichlids (derived from standing genetic variation, blue) and those that originated after the adaptive radiation (derived from a new mutation, orange). These origins were estimated by molecular phylogenetic analyses. Genes whose origins have not been examined because of a small number of informative SNPs in their coding regions are also shown (gray). (*c*) Comparison of maximum *d*_XY_ values in genes on highly differentiated regions. The separation is 1) genes which molecular phylogenetic analyses did not perform (gray), 2) those with alleles derived from standing genetic variation (blue), and 3) those with alleles derived from new mutation (orange). For the SNP data without SNP filtering based on HWE, we evaluated the differences in the distribution of maximum *d*_XY_ between genes whose origins are different by two-tailed Mann–Whitney test. ** indicates *P* < 0.001.

We calculated the weighted *F*_ST_ (Weir and Cockerham 1984) between each pair of species in sliding windows of 10 kb with 2-kb increments. The genomic regions with the top 0.5% of *F*_ST_ values for each distribution were designated as HDRs. We used strict criteria of the threshold *F*_ST_ because simulation analyses to directly test the effects of drift and selection for each HDR were difficult. Indeed, the population structures of three species were complex due to the existence of local subpopulations (supplementary fig. S2, Supplementary Material online) and possible hybridization with unknown populations (see discussion), which hampered the estimation of reliable population history used for simulation. The threshold *F*_ST_ for determining HDRs for each pair was 0.754386 for the pair consisting of *H. chilotes* and *H. sauvagei*, 0.584615 for that of *H. chilotes* and *L. rufus* and 0.671233 for that of *H. sauvagei* and *L. rufus*. Some HDRs showed species-specific differentiation, for example, HDRs between *H. chilotes* and *H. sauvagei* and between *H. chilotes* and *L. rufus* were designated as “*chilotes* specific.” In addition, to identify regions under strong positive selection, such as a selective sweep, we then calculated absolute measures of sequence differences per site (*d*_XY_) in sliding windows of 10 kb with 2-kb increments. The genome-wide pattern of genetic differentiation between species is shown in figure 4. The patterns associated with *F*_ST_ values are shown in figure 4*a*–*c*. In addition, figure 4*d*–*f* shows the plots of the two criteria for genetic differentiation, *F*_ST_ and *d*_XY_, in which the effects of selective sweep events can be observed. The red area of each plot indicates that the genomic region is highly differentiated in terms of the allele frequencies between species as well as the degree of sequence divergence. Interestingly, extremely high values of *d*_XY_ for HDRs tended to be observed in comparisons between *L. rufus* and the other two species, suggesting the footprints of strong positive selection in *L. rufus* (fig. 3*e* and *f*).

In total, we identified 678 candidate genes in HDRs including their 10-kb flanking regions (fig. 4*a* and supplementary table S3, Supplementary Material online). Among these candidate genes, 43 were *chilotes*-specific HDRs, 54 were *sauvagei*-specific HDRs and 63 were *rufus*-specific HDRs. We also investigated the allele frequencies of SNPs in candidate genes. The differentiated patterns of some candidate genes that we describe below are shown in supplementary figure S6, Supplementary Material online. The differentiated SNPs in HDRs were found in protein coding regions as well as in non-coding regions (putative cis-regulatory regions). The detailed inspection of these SNPs revealed that approximately 60% (401/678) of all candidate genes did not contain differentiated SNPs (i.e., with allele frequencies ≥ 0.5) in protein coding regions. Many differentiated SNPs were found in the introns or untranslated regions (UTRs). For example, inhibin beta B chain (*inhbb*) was located in a *chilotes*-specific HDR and had many differentiated SNPs that were present in introns under selective sweep (supplementary fig. S5*a*, Supplementary Material online). In addition, many differentiated SNPs were found in the UTR of 4-aminobutyrate aminotransferase (*abat*), which was located in a *rufus*-specific HDR (supplementary fig. S5*b*, Supplementary Material online).

We found differentiated SNPs on several genes of interest for which distinct expression patterns have been observed among cichlids in previous studies. For example, *rufus*-specific HDRs were found in intron 1 of Agouti-signaling protein 2 (*asip2b*) (supplementary fig. S5*c*, Supplementary Material online). Interestingly, mutations in a 1.1-kb intronic regulatory region of *asip2b* result in the loss of *asip2b* expression that leads to the loss of vertical stripes in Lake Victoria cichlids (Kratochwil et al. 2018; Urban et al. 2020). The presence of species-specific HDRs of *asip2b* in *L. rufus* is consistent with this study in that *L. rufus* do not possess vertical stripes, but the other two species do have such stripes. Cathepsin L (*ctsl*) located on HDRs between *H. chilotes* and *H. sauvagei* (supplementary fig. S5*d*, Supplementary Material online) is highly differentially expressed between *H. chilotes* and *H. sauvagei* (Kobayashi et al. 2006). The above two examples confirmed the validity of our methodology in terms of its accuracy and sensitivity with respect to characterizing actual HDRs that affect gene expression and the phenotypes of cichlids.

Based on gene ontology (GO) analyses for genes located on HDRs, we found candidate genes related to behavior, such as circadian entrainment, locomotory behavior and sensory system development (supplementary fig. S6, Supplementary Material online). Period circadian protein homolog 3 (*per3*) on a HDR between *H. chilotes* and *H. sauvagei* is related to circadian rhythms in behaviors and in metabolism in humans (Zhang et al. 2016). *Haplochromis chilotes* has several fixed polymorphisms in the exon and intron regions (supplementary fig. S5*e*, Supplementary Material online). Although we found no amino acid substitutions among these Lake Victoria cichlids, it is possible that a few fixed SNPs in intron regions may result in changes in gene expression in *H. chilotes*. SLIT and NTRK Like Family Member 6 (*slitrk6*) located on *chilotes*-specific HDRs (supplementary fig. S5*f*, Supplementary Material online) is associated with sensory perception, such as vision and hearing, via neural development in humans and mice (Tekin et al. 2013). Moreover, we found several copies of trace amine-associated receptors (*TAAR*s), which are members of the olfactory receptor gene family (Hashiguchi et al. 2008), in HDRs between *H. sauvagei* and *L. rufus* (supplementary fig. S5*g*, Supplementary Material online). Interestingly, such regions of *H. sauvagei* show obvious signatures of a selective sweep, implying that some of the *TAAR*s may contribute to species-specific adaptation by detecting species-specific chemical substances. We also found candidate genes related to the immune system, such as complement and coagulation cascades. For example, fibrinogen alpha chain (*fga*) and fibrinogen beta chain (*fgb*) were found in *rufus*-specific HDRs (supplementary fig. S5*h*, Supplementary Material online) and are involved in blood coagulation. Also, two tandem genes, transient receptor potential cation channel subfamily M member 4 (*trpm4*), in a *H. chilotes* and *H. sauvagei* HDR (supplementary fig. S5*i*, Supplementary Material online) are involved in immune responses based on the detection of a chemical stimulus via olfactory sensory neurons (Kastenhuber et al. 2013; Ellison et al. 2018; Sepahi et al. 2019). In addition to the above analyses, we also explored the HDRs without filtering the SNPs based on p-values of Hardy–Weinberg equilibrium (HWE, see Materials and Methods). As a result, 48 genes, including *LWS* (*opn1lw1*) and *RH1* (*rho*), were additionally identified to be located on HDRs.

### Genes with Divergent Alleles Derived from Ancient SGV

In cases where an allele of a gene derived from SGV is rapidly fixed within a population by a selective sweep, the differentiated haplotypes are expected to be highly divergent due to the accumulation of mutations during the long period of time from the split of the two alleles. In addition, theory predicts that the gene tree and the species tree become contradicting in cases where the divergent alleles are derived from SGV (Barrett and Schluter 2008). Therefore, to determine whether the genes with divergent alleles were derived from SGV, we constructed molecular phylogenetic trees among nine species of cichlids including examples from Lake Tanganyika, Malawi, and Victoria and riverine species for genes located on HDRs; we focused on those with a high *d*_XY_. Among 304 genes investigated, gene trees for 99 genes were inconsistent with the species tree, suggesting that the divergent alleles of these genes were derived from SGV that originated before the radiation of Lake Victoria (fig. 4*b*, table 1, supplementary fig. S7 and table S3, Supplementary Material online). Practically, the *d*_XY_ values for HDRs were significantly higher in genes with SGV than those with new mutations that originated before and after the radiation of Lake Victoria cichlids, respectively (fig. 4).

**Table 1.** Main Candidate Genes with Divergent Alleles Derived from Standing Genetic Variation.

HDR	NCBI Name	Allelic Origin^a^
*chilotes* specific	Cytochrome P450 2K1 [XP_014266774.1]	Before LT
*sauvagei* specific	Signal transducer and activator of transcription 1-alpha/beta [XP_003441521.1]	Before HB
	Phospholipid phosphatase-related protein type 4 [XP_005456534.1]	Before HB
*rufus* specific	Cadherin-related family member 5-like [XP_005454335.1]	Before HB
	Collagen alpha-6(VI) chain [XP_019219949.1]	Before HB
	Fibrinogen alpha chain [XP_013119750.1]	Before HB
	Fibrinogen beta chain [XP_003455581.2]	Before HB
	General transcription factor IIF subunit 2 [XP_005466907.1]	Before LT
*chilotes-sauvagei*	Cathepsin L1 [XP_003453258.1]	Before LT
	Transient receptor potential cation channel subfamily M member 4 [XP_019218187.1]	Before HB
*chilotes-rufus*	Collagen alpha-6(VI) chain [XP_019219953.1]	Before LT
	Intestinal mucin-like protein [XP_024659051.1]	Before LT
	Membrane cofactor protein [XP_025757175.1]	Before HB
*sauvagei-rufus*	Toll-like receptor 2 [XP_013119752.1]	Before LM
	TRPM8 channel-associated factor homolog [XP_014268336.2]	Before HB
	TRPM8 channel-associated factor homolog [XP_024660658.1]	Before HB

a Allelic origin indicates when the gene acquired divergent allele. LT, LM, and HB are the abbreviations of Lake Tanganyika, Lake Malawi, and *Haplochromis burtoni* from rivers near Lake Tanganyika, respectively.

**Fig. 5.**
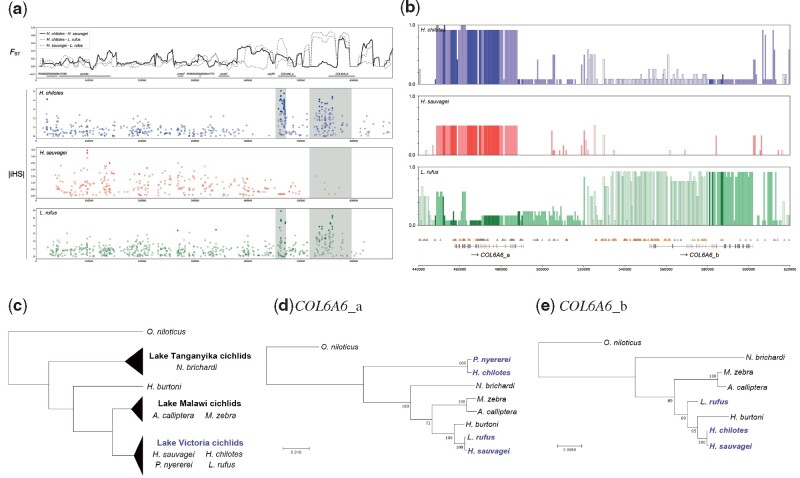
(*a*) Patterns of genetic differentiation on scaffold4484_cov51 where two paralogous Collagen alpha-6 (VI) chain (*COL6A6*) genes are located. First panel shows the degree of genetic differentiation between species based on the weighted mean of *F*_ST_ and the positions of representative genes. The values of *F*_ST_ were calculated for each 10-kb window. The three lower panels show absolute values for the integrated haplotype score (iHS) for each species. Extreme values of iHS, which are suggestive of selection, are indicated by the filled data points. Highly differentiated regions (HDRs) based on the *F*_ST_ values are shaded in gray. (*b*) Allele frequency of SNPs in the genomic region of two *COL6A6* genes. Vertical axis shows allele frequencies and horizontal axis shows the relative position of each SNP in the HDRs. Note that the allele frequencies of *Haplochromis sauvagei* do not exceed 0.5 because the SNPs were identified against the major alleles of *H. sauvagei*. Vertical bars of a deep shade represent the SNPs in exon regions, and those of a light shade indicate the SNPs in intron regions. Open vertical bars indicate the SNPs in the surrounding regions of two *COL6A6* genes. In the lowest plot, the triangles show the position of SNPs on the scaffold. In particular, SNPs exhibiting large differences in allele frequencies (≥0.6) between *H. chilotes* and *Lithochromis rufus* are highlighted in orange. (*c*) Species tree of East African Great Lakes cichlids based on Brawand et al. (2014). (*d*) Maximum likelihood estimated phylogenetic tree with 100 bootstrap replicates of *COL6A6_*a using nine cichlid species: *Oreochromis niloticus*, one Lake Tanganyika cichlid (*Neolamprologus brichardi*), a riverine species near Lake Tanganyika (*H. burtoni*), two Lake Malawi cichlids (*Astatotilapia calliptera* and *Maylandia zebra*) and four Lake Victoria cichlids (blue, *H. chilotes*, *H. sauvagei*, *L. rufus*, and *Pundamilia nyererei*). (*e*) Maximum likelihood estimated phylogenetic tree with 100 bootstrap replicates of *COL6A6_*b using eight of the cichlid species included in (*d*) (i.e., without *P. nyererei*). Note that some species in (*d*) and (*e*) were not included in the species tree because their genome sequences were fragmented due to assembly problems.

Collagen alpha-6 (VI) chain (*COL6A6*) was a striking example of the fixation of highly divergent alleles derived from SGV (fig. 5). We identified two paralogous *COL6A6* genes in cichlid genomes. We referred to the one in the upstream region as *COL6A6*_a (XP_019219953.1 collagen alpha-6(VI) chain isoform X1 [*Oreochromis niloticus*]) and the other in the downstream region as *COL6A6*_b (XP_019219949.1 collagen alpha-6(VI) chain isoform X1 [*O. niloticus*]). The extreme values of the integrated haplotype score (iHS) also indicated that many differentiated SNPs between *H. chilotes* and *L. rufus* were located on the two *COL6A6* genes (fig. 5*a*). Furthermore, both *COL6A6* genes had surprisingly long divergent haplotypes, consisting of >50 kb, which is a signature of a strong selective sweep (fig. 5*b*). For *COL6A6_*b, the haplotype belonging to the allelic type of *H. chilotes* and *H. sauvagei* can be discriminated from that of *L. rufus*. For *COL6A6*_a, *H. chilotes* and *L. rufus* were both mostly homozygous with different haplotypes. By contrast, *H. sauvagei* was not fixed to either of the differentiated haplotypes, suggesting that the allelic diversity of *COL6A6_*a has been retained in *H. sauvagei* (supplementary fig. S8, Supplementary Material online). Molecular phylogenetic analyses revealed that gene trees for both *COL6A6* divergent alleles contradicted the species tree in that the cichlids of Lake Victoria were not monophyletic (fig. 5*c*–*e*). Therefore, the divergent alleles observed in both *COL6A6* genes were derived from SGV.

## Discussion

In this study, we focused on two rocky-specialist species, *H. chilotes* and *H. sauvagei*, and one generalist species, *L. rufus*, that inhabit various lake bottom environments in Lake Victoria. We explored their species-specific genomic signatures for adaptation and its processes by inferring the history of these populations and by identifying candidate genes in HDRs. We found a substantial number of candidate genes in HDRs that contained long divergent haplotypes that originated before the radiation of Lake Victoria cichlids.

### Species-Specific Population History Depending on Ecological Backgrounds

Lake Victoria dried up approximately 14,600 years ago and then reappeared after a sudden rise in water level (Johnson et al. 2000). Such environmental changes would cause genetic admixture between populations that had been geographically separated, allowing them to gain new transgressive alleles. This event may have consequently facilitated the tempo of adaptation and thus is considered to be a trigger of adaptive radiation (Salzburger 2018). Although genetic introgression among Lake Victoria cichlids may have occurred after their adaptive radiation, no obvious signatures of introgression were observed among the three cichlids investigated in the present study. It is notable that our PCA analyses suggested that the two rocky-specialist species are likely to have population structure within species (supplementary fig. S2, Supplementary Material online). Significant differentiation between populations within rock-dwelling species was also found during mitochondrial and microsatellite analyses by Takeda et al. (2013). In addition, a previous morphological survey of *H. chilotes* reported the geographical variation in coloration (Seehausen 1996). Therefore, we expect that the two rocky-specialist species may have some locality-based subpopulations. We also found that the two rocky-specialist species have experienced population shrinkage, whereas the generalist species has retained its large population size. This result implies that differences in ecological backgrounds affected the pattern of changes in population size of these cichlids. However, it is necessary to confirm whether this interpretation applies to other species, with data that includes additional specialist and generalist species in future analyses.

Estimating the timing of speciation of Lake Victoria cichlids is a challenging issue due to the low degree of genetic differentiation among species. Our analyses showed that *H. sauvagei* initially diverged from the other two cichlids (fig. 2*c*), which is consistent with the results of Samonte et al. (2007) and Takeda et al. (2013). These results imply that *H. sauvagei* had already diverged from the other two cichlids before the adaptive radiation of Lake Victoria cichlids. Our estimated timing of divergence between *H. sauvagei* and the other two species (∼10,000 years ago) was somewhat more recent than a previous study (Samonte et al. 2007), in which the divergence was estimated to have occurred 41,300 years ago. The discrepancy may be due to differences in generation time and the mutation rate applied in the analysis. The population size of *H. chilotes* underwent a drastic decrease approximately 8,000 years ago based on the *smc++* analysis with the assumption that the generation time is 2 years (supplementary fig. S9, Supplementary Material online). We also obtained similar patterns by using *msmc2* analyses (fig. 2*d*), confirming the methodological validity of the estimation. Note that the estimated timing of speciation between *H. chilotes* and *H. sauvagei* was similar to the time at which a decline in the size of their populations occurred. It is possible that the acquisition of new habitats due to changes in the water level may have led to the formation of small founder populations that were geographically separated, thus facilitating local adaptation (Salzburger 2009). Therefore, each of the ancestral populations of the two rocky-specialist species (*H. chilotes* and *H. sauvagei*) may represent examples of adaptation from small founder populations.

### Genes That May Have Contributed to Adaptation of Lake Victoria Cichlids

In this study, we identified 678 candidate genes, some of which are interesting for further discussion about their possible functional roles. For example, the gene encoding Cathepsin L (*ctsl*) located on HDRs from the *H. chilotes* and *H. sauvagei* comparison is highly expressed in the jaw of 15-day fries of *H. chilotes* as compared with *H. sauvagei* (Kobayashi et al. 2006). Given that Cathepsin L is secreted and is involved in degradation of extracellular matrix proteins, such as collagens, elastin, and other structural proteins (Kirschke 1998; Mort 1998), it may play an important role in the distinct species-specific morphology of the jaw and lip that has been observed between *H. chilotes* and *H. sauvagei*. Namely, it is possible that the higher expression of Cathepsin L during development results in the hypertrophied lip of *H. chilotes*. The *TAAR* genes located on HDRs from the *H. sauvagei* and *L. rufus* comparison are also interesting candidates for adaptation. Although biologists have paid less attention to olfaction than to vision with respect to mechanisms of adaptation and speciation, several recent studies propose the substantial contribution of olfaction in the reproductive communication of cichlids (Plenderleith et al. 2005; Cole and Stacey 2006). Indeed, Keller-Costa et al. (2014) identified urinary pheromones released by dominant males of tilapia that induce reproduction in females. The existence of divergent alleles of *TAAR*s that belong to one of the olfactory receptor multigene families suggests the possibility of speciation and/or adaptation due to a difference in the ability to detect particular chemical substances.

It is notable that a large fraction of the divergent alleles in HDRs was derived from SGV. Lake Victoria cichlids possess divergent alleles that are shared among cichlids of other East African Great Lakes. A striking example is the dimorphic allelic diversity of *V1R2*, which is also present in cichlids from Lakes Malawi and Tanganyika (Nikaido et al. 2014). The dimorphic allelic diversity in *LWS* of Lake Victoria cichlids also predates their radiation (Terai et al. 2002; Seehausen 2004). In Lake Victoria cichlids, the divergent allele groups have allowed populations to adapt to differences in habitat turbidities and have given rise to diversity in male nuptial coloration (Terai et al. 2006). In contrast, the reduced diversity among populations in Lakes Malawi and Tanganyika may be due to the consistent transparency of these two lakes. We found that *L. rufus* had the divergent alleles of *LWS* and *RH1*, which were not classified to the known allele groups of Lake Victoria cichlids (Terai et al. 2006; Miyagi et al. 2012; Terai et al. 2017) (supplementary fig. S10, Supplementary Material online). Although our analyses did not show that both genes had divergent alleles derived from SGV, these novel alleles may have the important roles on mate recognition of *L. rufus*, which has noticeable red nuptial coloration in male. In addition, Takuno et al. (2019) showed that the origins of gene variants in 16 differentiated regions predate the adaptive radiation of Lake Victoria cichlids. Two of the genes among these 16 regions, intestinal mucin-like protein (*ENSMZEG00005001611*) and general transcription factor IIF subunit2 (*gtf2f2a*), were also identified in our study as HDRs with divergent alleles derived from SGV (supplementary fig. S5*k* and *l*, Supplementary Material online).

In our study, 99 genes with highly divergent alleles derived from SGV were identified as strong candidates contributing to the adaptation of Lake Victoria cichlids (table 1, fig. 4*b*). The candidate genes cover a wide variety of ecological functions, such as circadian rhythm, locomotion, and sensory systems, which may provide important insights into understanding the process of environmental adaptation. Notably, two *COL6A6* genes, in which the signature of a strong selective sweep was detected over a 50-kb region, are of scientific interest. Collagen VI is a major extracellular matrix protein and functions in the development of skeletal muscle, maintenance of skin integrity and the immune system (Cescon et al. 2015). Zebrafish have two paralogous *COL6A6* genes on different chromosomes, whereas many species which belong to Euteleostei contain two paralogous *COL6A6* genes in tandem in one genomic region, suggesting that gene duplication of teleost *COL6A6* genes occurred repeatedly during teleost evolution (Ramanoudjame et al. 2015; Tonelotto et al. 2019). Our phylogenetic tree of *COL6A6* genes revealed that the tandemly duplicated paralogs of *COL6A6_*a and *COL6A6_*b are each monophyletic (supplementary fig. S11, Supplementary Material online). Importantly, gene trees of both *COL6A6_a* and *COL6A6_b* of East African cichlids were inconsistent with the species tree implying that the alleles were derived from SGV (fig. 5*c*–*e*). It is also noteworthy that the two divergent alleles of *COL6A6_*a have been maintained at the subpopulation level in *H. sauvagei*, suggesting the signature of recombination suppression across a 30-kb region (fig. 5*a* and *b*, supplementary fig. S8, Supplementary Material online). In addition, *COL6A6* genes have been listed as candidate genes for adaptation on HDRs between two rocky species in Lake Victoria, *P. pundamilia* and *P. nyererei* (Meier et al. 2018), although the existence of two paralogs and detailed allelic structures have not yet been identified in these two species. The findings indicate the possibility that divergent alleles in *COL6A6_*a and *COL6A6_*b have been repeatedly recruited in cichlids through natural selection according to changes in the environment caused by biotic and/or abiotic factors.

In this study, we used only three species of Lake Victoria cichlids, which comprise more than 500 species, because enough individuals for each species needed to be included to accomplish the population genetic analyses at the whole-genome level. As the number and combination of species compared in the analyses increases, it is highly likely that more genes with SGV will be detected. For example, on the molecular phylogenetic analysis of *asip2b*, which is responsible for the phenotypic divergence of vertical stripe patterns, we did not observe the existence of divergent alleles derived from SGV. However, Urban et al. (2020) showed that the origins of divergent alleles in Lake Victoria cichlids had predated to the radiation, by whole-genome comparative analyses of 22 species (36 individuals) including those from the surrounding lakes of Lake Victoria. In our analyses, the origins of genes on HDRs with lower values of *d*_XY_ were not examined because the elucidation of reliable phylogenetic trees was made difficult by the insufficient number of informative SNPs that had accumulated (fig. 4*b* and *c*). Although many of these genes are considered to have divergent alleles derived from new mutations, it is likely that a substantial number of these genes may have divergent alleles from SGV when selection has worked against only a few variants whose origins predate the adaptive radiation of Lake Victoria cichlids. Furthermore, the expression patterns of the genes on HDRs with lower values of *d*_XY_, including those that have divergent alleles from new mutations, may be regulated by cis- or trans-regions with divergent alleles from SGV. Thus, the genes we found in the present study may represent just a small portion of the genomic factors that facilitated the rapid adaptation of Lake Victoria cichlids. Such large-scale SGV could use suitable alleles at moderate frequencies within species when the environment changes, resulting in mosaic genomes (Salzburger 2018). Our findings in this study re-emphasize the genomic complexity involved in the adaptive radiation of Lake Victoria cichlids.

### Complexity of the Genomic Substrate for Adaptation of *L. rufus*

*Lithochromis rufus*, a generalist species, showed the highest level of genetic diversity within species as compared with the two rocky-specialist species, in which population structures were observed according to their localities. One possible explanation for the high level of genetic diversity in *L. rufus* is the formation of a metapopulation. In a metapopulation, the genetic population is maintained at a larger size than expected because occasional genetic admixtures occur among genetically distant populations. Furthermore, the higher rate of heterozygosity in *L. rufus* suggests the possibility of hybridization with unknown populations or even species that are genetically distant. Previous studies showed that *L. rufus* and some pelagic species in Lake Victoria are genetically close (Takeda et al. 2013). In addition, *L. rufus* had more HDRs with a higher number of SNPs as compared with the other two species (fig. 3*e* and *f*). Most of the genes located on such HDRs in *L. rufus* possess divergent alleles derived from SGV. Previous studies have suggested the importance of introgression among species with respect to the adaptive radiation of cichlids (Meier et al. 2017, 2019; Malinsky et al. 2018; Svardal et al. 2020). The results of our study indicate the possibility that *L. rufus* obtained transgressive alleles by introgression from pelagic species in Lake Victoria and/or from riverine species, which enabled the adaptation of a generalist species, *L. rufus*. To determine in more detail the process of adaptation that *L. rufus* underwent, it is necessary to explore and specify the unknown population that provided the genetic resource for the adaptation of *L. rufus*. Estimation of the population history with a model-based method with genomic data from tens of individuals is also necessary. The unique and complex evolutionary process of *L. rufus* provides a great opportunity for understanding species diversity derived from the complexity of genomic substrates that facilitated the adaptation of Lake Victoria cichlids.

## Conclusions

Genome-wide comparative analyses of three Lake Victoria cichlids enabled us to uncover the processes of species-specific adaptation. We showed that *H. sauvagei* initially diverged from other cichlids and that some species were still under ongoing adaptation to each locality. Moreover, the patterns of changes in population size were quite distinct among the species according to the ecological backgrounds. The exploration of HDRs among species provided many candidate genes contributing to adaptation. Molecular phylogenetic analyses for candidate genes on HDRs revealed that the allelic diversities were derived from SGVs, which originated before the adaptive radiation. Thus, our findings in this study highlight the substantial contribution of large-scale SGVs to the rapid adaptations of Lake Victoria cichlids.

## Materials and Methods

### Samples and Sequencing

We collected six wild males of each species, *H. chilotes* (Boulenger 1911), *H. sauvagei* (Seegers 2008), and *L. rufus* (Seehausen et al. 1998), from Mwanza Gulf and its surroundings in Lake Victoria for our analyses (fig. 1*b* and supplementary table S1, Supplementary Material online). *H. chilotes*, one of the most widely distributed species among Lake Victoria rock-dwelling cichlids, is characterized by its thick lips. *H. chilotes* inhabits predominantly rocky shores near sandy areas in shallow to deep water. *H. chilotes* is an omnivorous species and feeds on mainly prawn and sometimes insect larvae by pressing its lips to cracks between rocks to suck out the larvae (Greenwood 1959). *Haplochromis sauvagei* inhabits predominantly rocky shores in shallow water. *Haplochromis sauvagei* is an omnivorous species and feeds on predominantly insect larvae and algae on rock surfaces. Characteristics that are shared by these two species are the chessboard-like patterns on their bodies and their habitats, which are mainly restricted to rocky shores (rocky-specialist). *Lithochromis rufus*, an endemic species to Mwanza Gulf, is characterized by the bright red male nuptial coloration (Seehausen et al. 1998). Although *L. rufus* has been described as a rock-dwelling species, our survey caught individuals not only in rocky areas but also in sandy areas and vegetation zones, showing that *L. rufus* is a generalist with respect to its habitat. *Lithochromis rufus* is an omnivorous species and feeds on insect larvae and tiny snails (Seehausen et al. 1998). Sampling points for each species were Nyegezi (*n *=* *1), Makobe Island (*n *=* *1), Kilimo Island (*n *=* *1), and Chamagati Island (*n *=* *3) for *H. chilotes*; Nyegezi (*n *=* *1), Bwiru Peninsula (*n *=* *1), and Chamagati Island (*n *=* *4) for *H. sauvagei* and Nyegezi (*n *=* *1), Kilimo Island (*n *=* *2) and Nyaruwambu (*n *=* *3) for *L. rufus* (fig. 1*b*). Detailed information for each individual is shown in supplementary table S1, Supplementary Material online. DNA samples were extracted from a fin clip of each individual and sequencing. For all samples, we prepared Illumina paired-end libraries using TruSeq DNA PCR-Free LT Sample Prep Kit and performed sequencing using an Illumina HiSeq 2500.

### De Novo Assembly of the Draft Genomes

We selected one *H. chilotes* individual (ID number = 16633, HC02 in supplementary table S1, Supplementary Material online), one *H. sauvagei* individual (ID number = 17621, HS08 in supplementary table S1, Supplementary Material online) and one *L. rufus* individual (ID number = 17820, LR18 in supplementary table S1, Supplementary Material online) to construct the draft genomes. For these samples, mate-pair libraries were additionally prepared using Nextera Mate Pair Sample Prep Kit. Sequencing was performed using an Illumina HiSeq 2500. The statistics of the generated reads are shown in supplementary table S2, Supplementary Material online. The draft genomes of *H. chilotes*, *H. sauvagei* and *L. rufus* were constructed through *de novo* assemblies. For each sample, paired-end and mate-pair libraries were assembled as follows. 1) Paired-end and mate-pair reads were preprocessed using platanus_trim and platanus_internal_trim (version 1.0.7; http://platanus.bio.titech.ac.jp/), respectively, to exclude adaptor sequences and low-quality bases with default parameters. 2) Inputting only paired-ends, Platanus (version 1.2.4) (Kajitani et al. 2014) was executed with the “assemble,” “scaffold,” and “gap_close” commands designated by default parameters. 3) Short-insert reads (estimated insert size ≤ 0.5 × nominal size) and PCR duplicates in mate-pair libraries were removed based on mapping of reads to the assembly results (scaffolds), which were constructed only from paired-end libraries, using an in-house program. The mapping was performed using Bowtie2 (version 2.1.0) (Langmead and Salzberg 2012) as the single-end mode. 4) The “scaffold” and “gap_close” commands of Platanus were re-executed with default parameters after inputting the contigs (the result of the “assemble” command in (2)) and paired-ends and mate-pairs processed in (3). 5) From the resulting scaffolds, short ones (length, <500 bp) and contamination candidates that matched bacterial or viral genomes were excluded; the contamination candidates were determined based on NCBI Bacteria and RefSeq viral genomes databases with BlastN (version 2.2.26) (Camacho et al. 2009) with the threshold identity ≥90% and query-coverage ≥50%. The statistics of the final assemblies are shown in supplementary table S2, Supplementary Material online.

### Mapping and Variant Calling

After the ascertainment of the quality of reads by FastQC (Andrews 2010), raw reads were mapped to the de novo assembly genome of *H. chilotes* (N50 = 1,540,223 bp), which was designated as the reference genome, for each individual using *bwa-mem* (version 0.7.17-r1188) (Li 2013). Before this step, we detected such regions on our reference genome using RepeatMasker (Churakov et al. 2005) and then masked them by overwriting N to moderate the effects of repetitive sequences on further analyses. We obtained only unique reads with reasonable mapping quality using option “-f 2 -F 2052 and -q 30” in *samtools* (version 1.8) (Li et al. 2009). The average mapping coverage ranged from 19.2 (*H. sauvagei* individual from Bwiru Peninsula, HS07) to 30.8 (*H. chilotes* individual from Chamagati Island, HC05) (supplementary table S1, Supplementary Material online). Variant calling against the reference genome was performed with *samtools* and *bcftools* (version 1.8) (Li 2011). To avoid the effects of mapping errors, we allowed only SNPs with a minimum depth = 10, maximum depth = 60 and minimum mapping quality = 60 for each individual using “vcffilter” command in *vcflib* (Garrison, 2016) and the option “--minQ” in *vcftools* (Danecek et al. 2011). After merging data for all individuals, we extracted only biallelic sites, trimmed all sites with missing data using the option “--max-missing 1” and then removed all insertions and deletions using the option “--remove-indels” in *vcftools*. Also, we excluded sites that deviated from HWE (*P* < 0.001) using the option “--hwe 0.001” in *vcftools*. Finally, we obtained 1,879,895 biallelic SNPs for further analyses.

### Statistics

To evaluate nucleotide diversity within species and levels of genetic differentiation between pairs of species, we subdivided the genome-wide SNP data set into 10-kb windows with 2-kb increments along each scaffold with a sliding window approach. By using *vcftools*, nucleotide diversity (*π*) was calculated for windows of each species, and the weighted mean of *F*_ST_, which is a relative measure of the genetic distance between species (Weir and Cockerham 1984), was calculated for windows of each pair of species. Absolute sequence differences between populations (*d*_XY_) were also calculated for windows of each pair of species by *PopGenome* (Pfeifer et al. 2014). To assess the degree of heterozygosity among genome-wide SNP data for each individual, we also calculated the inbreeding coefficient using *vcftools*.

### Population Structure Analyses

For population structure analyses, we selected SNPs with minor allele frequency ≥0.05 based on *vcftools*. Then, we extracted LD-pruned SNPs in plink format under linkage equilibrium (LD) using the option “--indep-pairwise 50 5 0.1” in *plink* (version 1.9) (Purcell et al. 2007). Finally, we obtained a population structure analyses data set with 61,461 LD-pruned SNPs in vcf format. For the PCAs, we generated input data from our data set using *PGDSpider* (version 2.1.1.5) (Lischer and Excoffier 2012) and ran the smartpca program in *EIGENSOFT* software (Patterson et al. 2006) with default parameters. Also, we ran ADMIXTURE (Alexander et al. 2009), a program that carries out model-based ancestry estimation using maximum likelihood, from *K *=* *1 to *K *=* *6.

### Inference of Population History over Generations

Because SNPs under selection give some bias with respect to the quality of inference of population history (Ewing and Jensen 2016), we selected the 383 largest scaffolds (≥500 kb) without regions that included both the top 0.5% values of the distribution of *F*_ST_ and the top 0.5% values of the distribution of *d*_XY_; these scaffolds accounted for 73.7% of our *H. chilotes* reference genome. First, the pattern of changes in population size was inferred by the multiple sequential Markovian coalescent program MSMC (Schiffels and Durbin 2014). We generated input data from bam files with only unique reads following the criteria on http://github.com/stschiff/msmc-tools without the reference panel. SNPs were included if their depths were within the range of half of the average for their region to double that average. Haplotype phasing was performed by BEAGLE 4 (version 4.1) (Browning and Browning 2007). To generate mappability data, we used SNPable (http://lh3lh3.users.sourceforge.net/snpable.shtml). We divided the reference genome into overlapping *k*-mers (we used *k *=* *50), and these were aligned back to our reference genome based on the method of Malinsky et al. (2018). For each analysis, we ran the program *msmc2* (Malaspinas et al. 2016) with 100 iterations using data from four haplotypes (two individuals) selected from the six individuals for each species. In addition, the pattern of changes in population size for each species and the timing of population splits between species were inferred by *smc*++ (Terhorst et al. 2017). By using vcf2smc, input data were generated from 12 unphased haplotype data from each species for each scaffold. This scaffold set in smc++ analyses was the same set as *msmc2* analyses. Because of our small sample size, we divided the scaffold data into chunks of 500 kb each. Then, we ran *smc*++ with 100 bootstrap replicates. For all analyses, we used 3.5 × 10^−9^ mutations per base pair per generation as the mutation rate (Malinsky et al. 2018).

### Identification of Genes in Highly Differentiated Genetic Regions

To identify genetic regions under selection, we classified genetic regions with the top 0.5% of values for the *F*_ST_ distributions of each pair of species as being HDRs. In addition, we also computed the iHS for each SNP (Voight et al. 2006) using selscan (version 1.2.0a) (Szpiech and Hernandez 2014) with default parameters against phased whole-genome data. Phasing was performed by BEAGLE 4 (version 4.1) (Browning and Browning 2007). We used the program norm (version 1.2.1a) (Szpiech and Hernandez 2014) with the option “--crit-percent 0.01” to identify SNPs under selection, that is, those showing extreme iHS values. Next, we checked whether such HDRs contained certain genes against genome assembly data of *O. niloticus* (Orenil1.0 or O_niloticus_UMD_NMBU) and *Maylandia zebra* (M_zebra_UMD2a) using BlastN searches in Ensembl (Cunningham et al. 2019). To double-check the presence of candidate genes, we further executed local TBlastN searches with BLAST+ (Camacho et al. 2009) against the *H. chilotes* genome assembly data using amino acid sequences of candidate genes from the NCBI database (https://blast.ncbi.nlm.nih.gov) as query sequences. We also identified UTRs by BlastN searches. For all candidate genes, we automatically predicted exon regions using GeneWise (Birney et al. 2004) at EMBL-EBI (https://www.ebi.ac.uk) and determined in which gene regions divergent SNPs were located. GO enrichment analyses were performed by WebGestalt (Liao et al. 2019). We chose “gene ontology” and “pathway” categories as functional database and investigated the functions of candidate genes based on “protein-coding data” from the human genome. We did not use genes that did not have a corresponding human gene in these analyses.

### Estimation of the Origins of Divergent Alleles

To explore adaptive alleles derived from SGV, we examined whether the alleles were shared across Lake Victoria cichlids. We obtained available genome assembly data for *O. niloticus* (from rivers across northern Africa, Orenil1.0), *Neolamprologus brichardi* (from Lake Tanganyika, NeoBri1.0), *H. burtoni* (from rivers near Lake Tanganyika, AstBur1.0), *Astatotilapia calliptera* (from Lake Malawi, fAstCal1.2), *M. zebra* (from Lake Malawi, M_zebra_UMD2a), and *P. nyererei* (from Lake Victoria, PunNye1.0) from the Ensembl database. Whole-genome sequences of five of these species (with the exception of *A. calliptera*) were determined by Brawand et al. (2014). Additionally, we also used de novo-assembled genome data from *H. chilotes*, *H. sauvagei*, and *L. rufus* from Lake Victoria. Against each set of assembled genome data, we executed local TBlastN searches with BLAST+ using amino acid sequences of candidate genes from the NCBI database (https://blast.ncbi.nlm.nih.gov) as query sequences. We then identified coding regions in the genomic data for each species using GeneWise (Birney et al. 2004). Across the sequences of exon regions, we performed multiple sequence alignments at the amino acid level with default parameters with ClustalW/MUSCLE in MEGA7 (Kumar et al. 2016). We also generated alignments for nucleotide sequences of gene regions including intron regions and UTRs. Finally, we obtained a set of sequence alignments for molecular phylogenetic analyses, which comprised 1) amino acid sequences of exon regions, 2) nucleotide sequences of exon regions, and 3) nucleotide sequences of gene regions. For each sequence alignment, we constructed maximum-likelihood phylogenetic trees with 100 bootstrap replicates on default parameters of MEGA7 (Kumar et al. 2016). *Oreochromis niloticus* was chosen as the outgroup. We used sites without deletions for phylogenetic analyses. Candidate genes for which their phylogenetic tree of the four Lake Victoria cichlid species did not result in a monophyletic tree and that had a bootstrap value ≥60 were considered as having divergent alleles derived from SGV. Note that a species was excluded from the phylogenetic tree construction in cases where its corresponding gene sequence was fragmented due to assembly problems. For these analyses, we cannot ignore the possibility that highly differentiated genes with divergent SNPs under strong selection were missed by SNP filtering based on the degree of the deviation from HWE. Therefore, we further performed similar analyses against the genes on newly designated HDRs without SNP filtering based on HWE.

As for the two *COL6A6* genes, we conducted further analysis including the genomes of three Ostariophysi species, *Danio rerio*, *Astyanx mexicanus*, and *Pygocentrus nattereri*, with *Oryzias latipes* and *Lepisosteus oculatus* as the outgroups. These sequences were obtained from the NCBI database (https://blast.ncbi.nlm.nih.gov). We constructed the maximum-likelihood phylogenetic tree for the *COL6A6* genes with 100 bootstrap replicates.

### Determination of the Alleles of Focal Genes

As for divergent alleles of *COL6A6*_a, *LWS* and *RH1*, we investigated the allelic type of each site per haplotype from SNP data. Previous studies determined several kinds of *LWS* and *RH1* allele in Lake Victoria cichlids and nonsynonymous substitutions on a triallelic site have been observed in the *LWS* allele groups (Terai et al. 2006, 2017; Miyagi et al. 2012). To identify the divergent alleles of *LWS* and *RH1* in our study by comparing with previous results, we used the multiallelic SNP data which allowed all sites with missing data and those with the deviation from HWE. Based on the SNP data, we determined the haplotype using the option “consensus” in *bcftools* (Li 2011) and we automatically predicted exon regions using GeneWise (Birney et al. 2004) at EMBL-EBI (https://www.ebi.ac.uk).

## Supplementary Material

Supplementary data are available at *Molecular Biology and Evolution* online.

## Supplementary Material

msab084_Supplementary_Data
