# Supplementary material for: Genomic Signatures for Species-Specific Adaptation in Lake Victoria Cichlids Derived from Large-Scale Standing Genetic Variation
Source: Mol Biol Evol. 2021 Mar 21;38(8):3111–25. doi: 10.1093/molbev/msab084 (PMC8321545; doi:10.1093/molbev/msab084)
Supplement: msab084_Supplementary_Data [file msab084_supplementary_data.zip › supplementary_figure.pdf]

**Supplementary Figures**

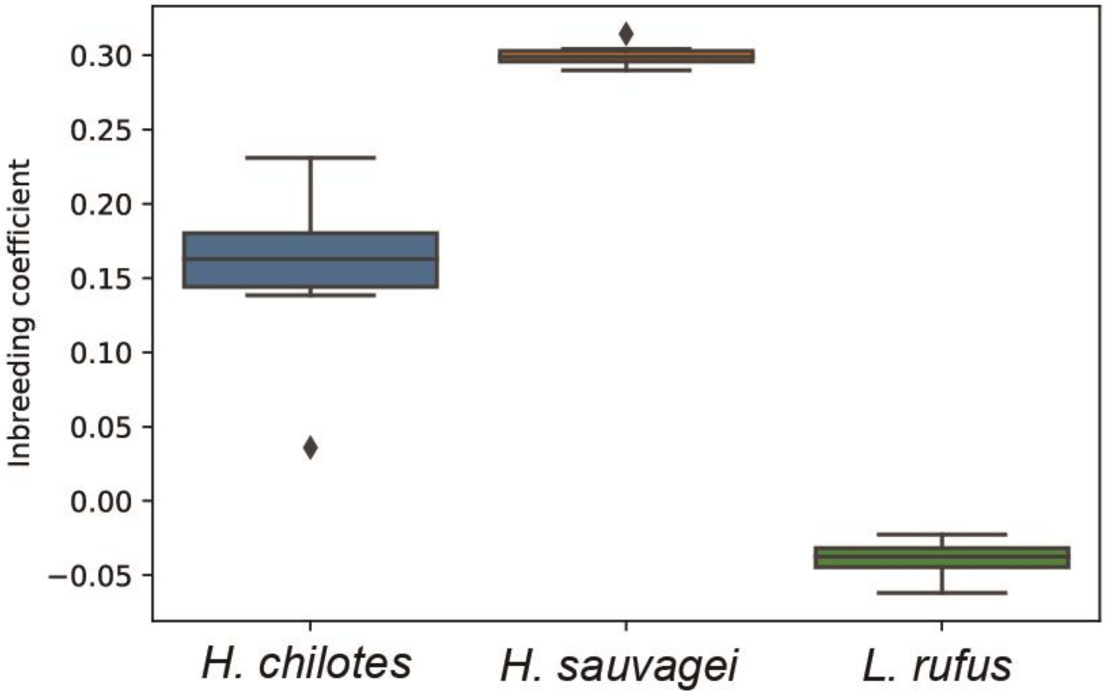


**Fig. S1.** Inbreeding coefficient of each species. The values were obtained by calculating them for each individual. Negative values show excess of heterozygosity throughout the genome.

(a)

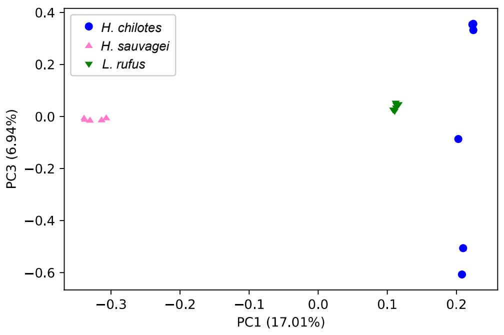


(b)

| Individual name | Sampling Point | PC3 |
|---|---|---|
| HC01 | Nyegezi | -0.5063 |
| HC02 | Chamagati Island | 0.3538 |
| HC03 | Makobe Island | -0.0866 |
| HC04 | Kilimo Island | -0.6074 |
| HC05 | Chamagati Island | 0.3564 |
| HC06 | Chamagati Island | 0.3321 |

**Fig. S2.** PCA of genetic variation for all individuals of *H. chilotes*, *H. sauvagei* and *L. rufus*. Each plot explains the genetic structure for each individual. (a) PC1 and PC3. (b) Values of PC3 of *H. chilotes* individuals and the sampling points.

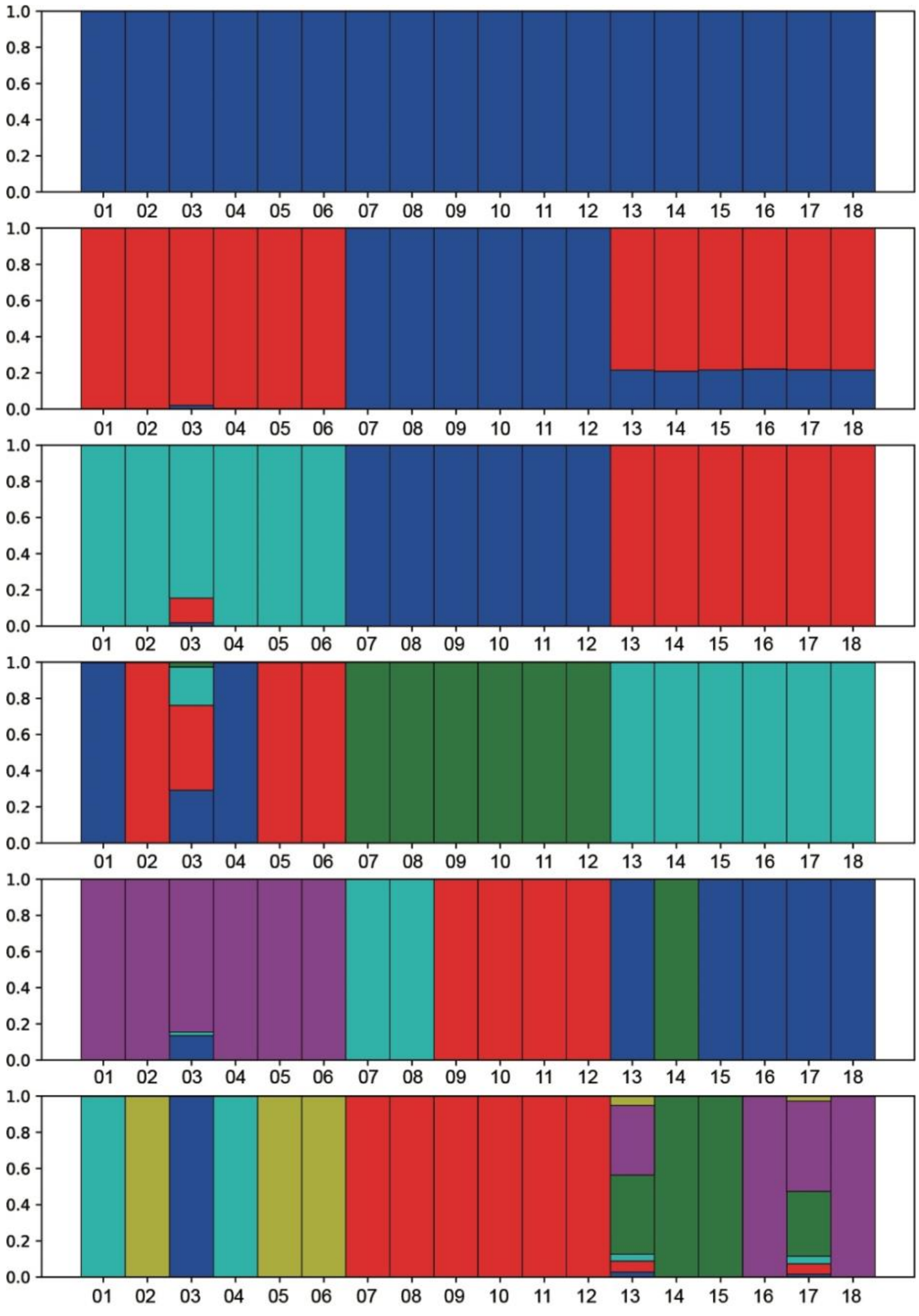


**Fig. S3.** Results of ADMIXTURE analyses of entire individuals with $K$=1-6. For these analyses, 61,461 LD-pruned SNPs are used. Results of *H. chilotes* are from 01 to 06, those of *H. sauvagei* are from 07 to 12, and those of *L. rufus* are from 13 to 18.

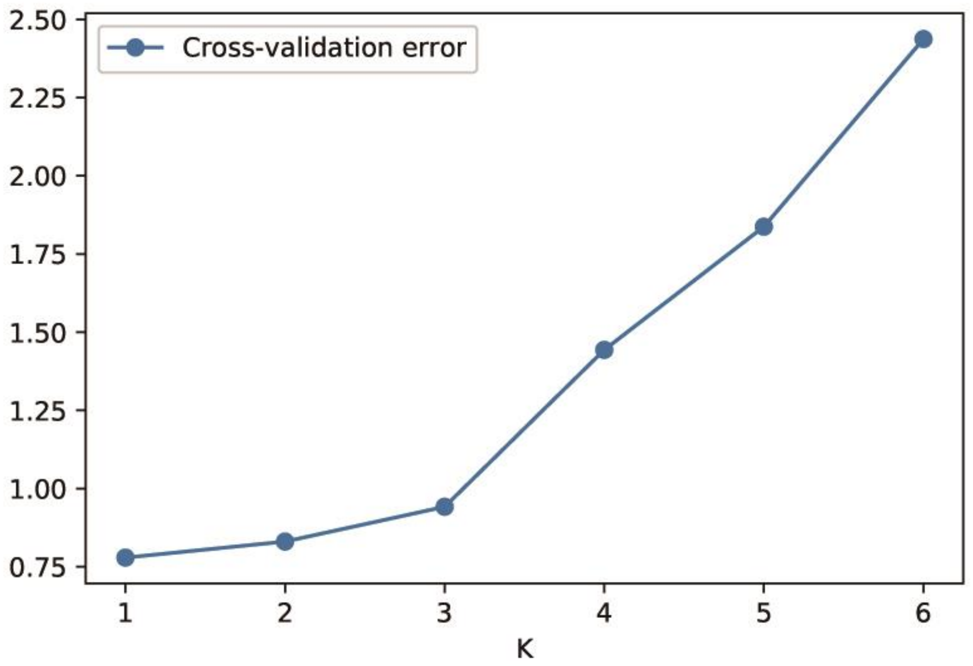


**Fig. S4.** Cross-validation error rate of each result of ADMIXTURE analyses. The rates were obtained from ADMIXUTRE analyses which are shown in fig. 2 and supplementary fig. S3, Supplementary Material online.

(a) *inhbb* (XP_003445380.1 inhibin beta B chain [Oreochromis niloticus])

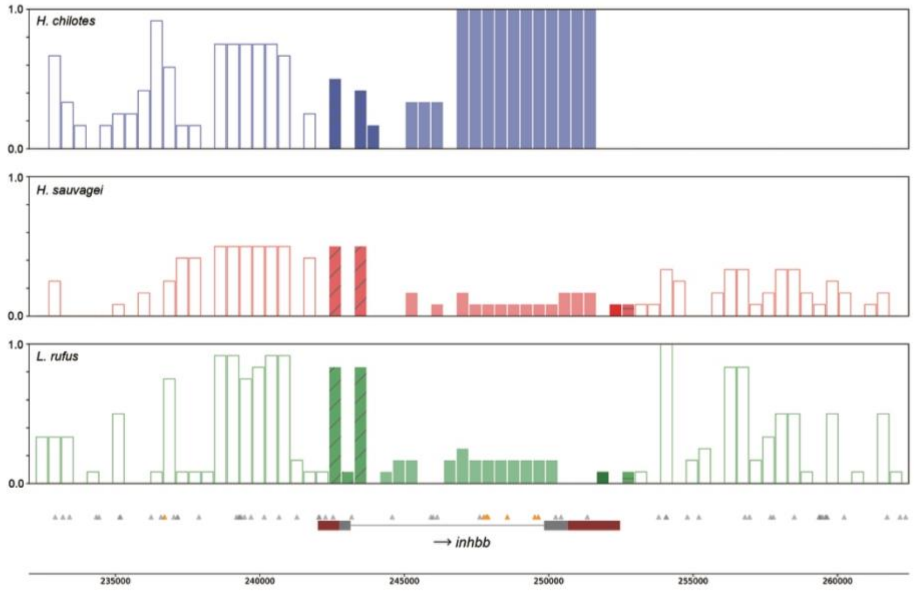


(b) *abat* (XP_025762502.1 4-aminobutyrate aminotransferase, mitochondrial isoform X1 [Oreochromis niloticus])

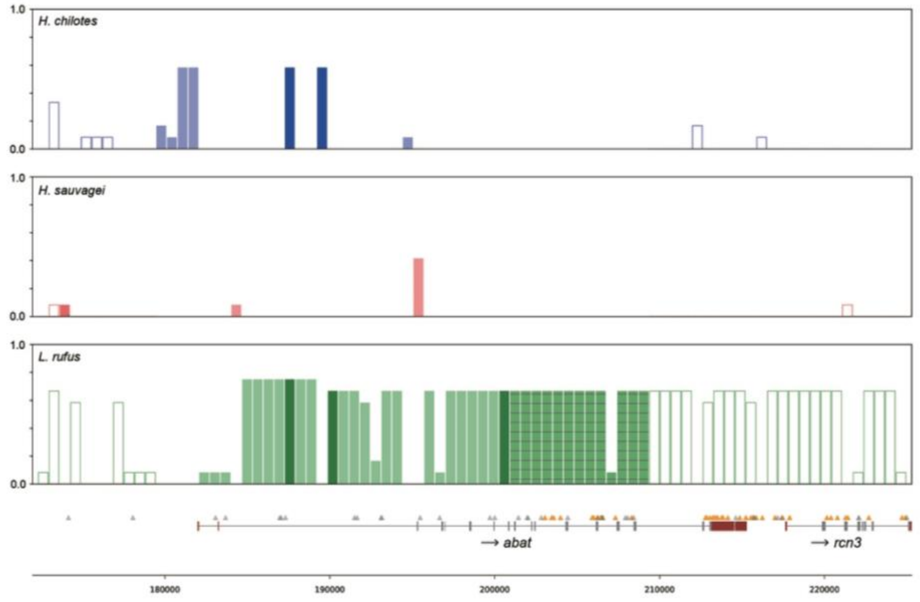

(c) *asip2b* (XP_005476024.1 agouti-related protein-like isoform X1 [Oreochromis niloticus])

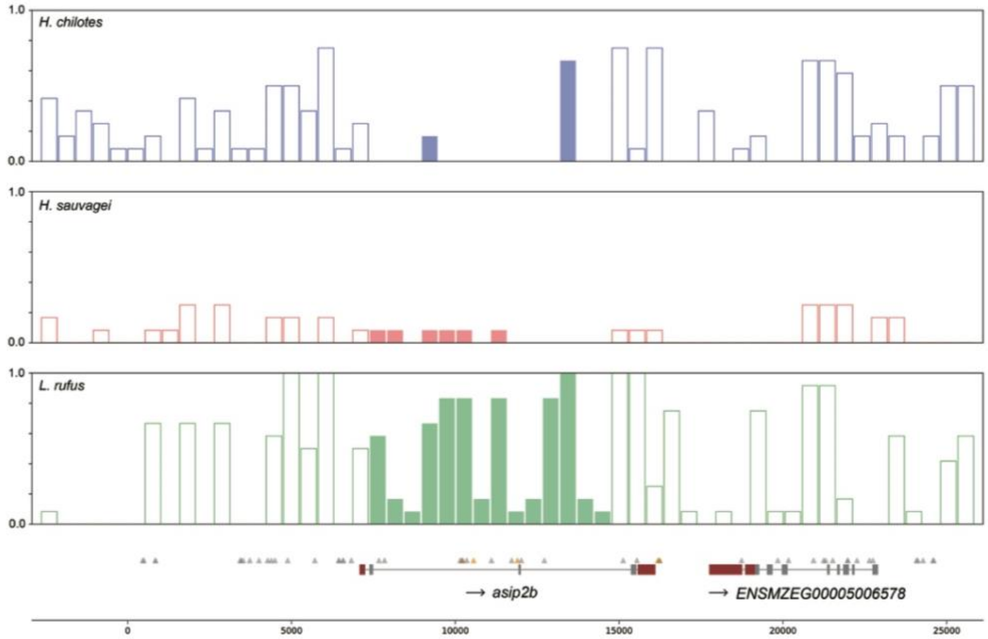


(d) *ctsl* (XP_003453258.1 cathepsin L1 isoform X1 [Oreochromis niloticus])

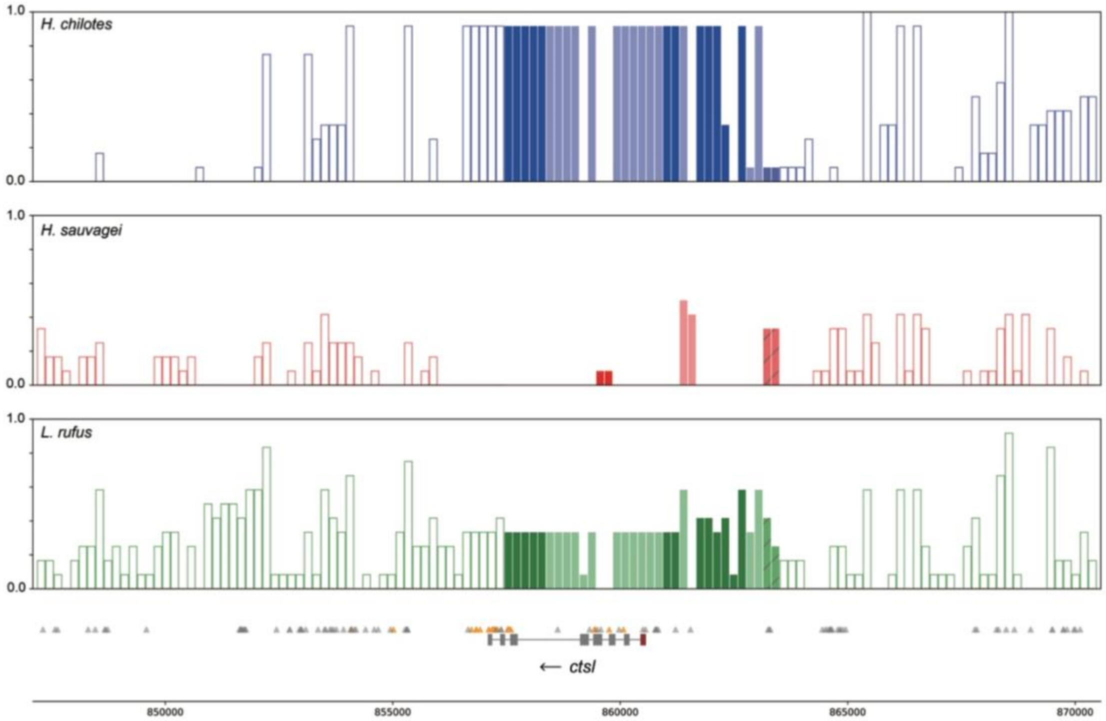

(e) *per3* (XP_005469396.2 period circadian protein homolog 3 isoform X1 [Oreochromis niloticus])

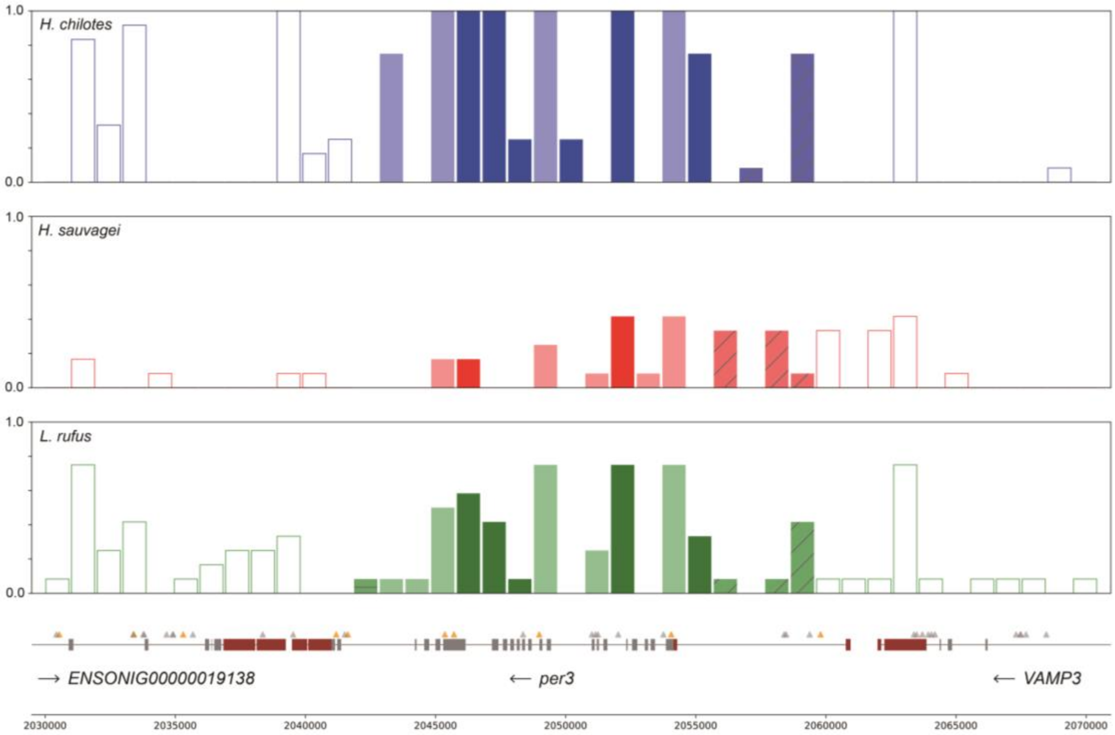


(f) *slitrk6* (XP_005450063.1 SLIT and NTRK-like protein 6 [Oreochromis niloticus])

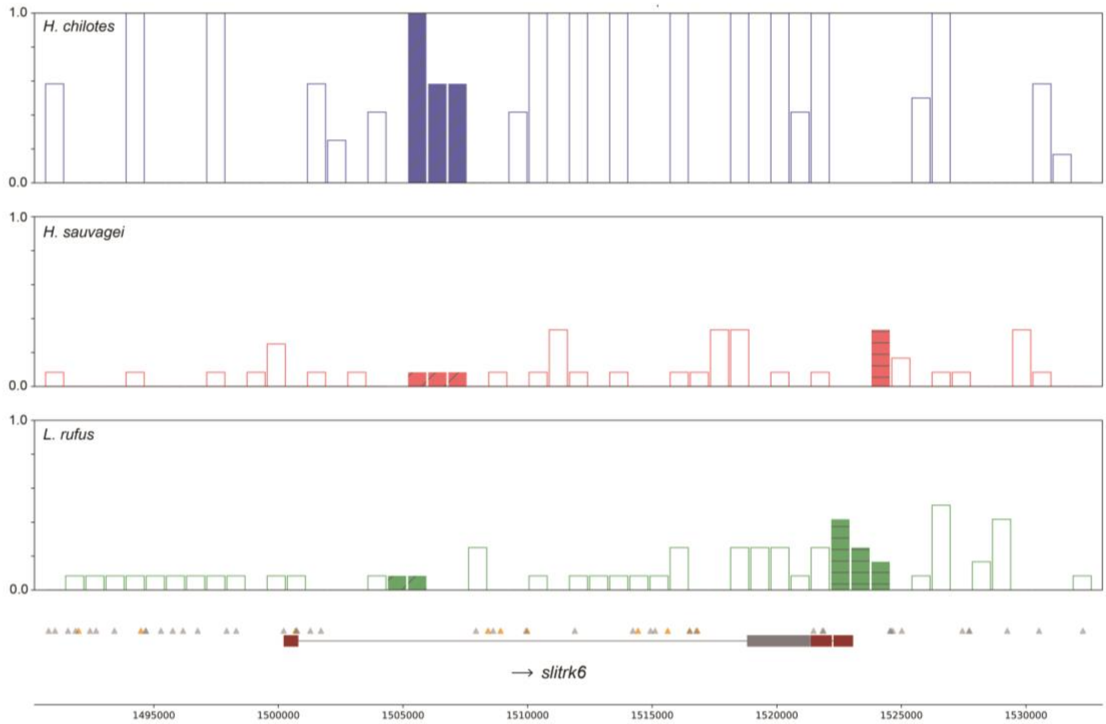

(g) *TAAR*s

*ENSMZEG00005028047* (XP_005475358.4 trace amine-associated receptor 13c-like [Oreochromis niloticus])

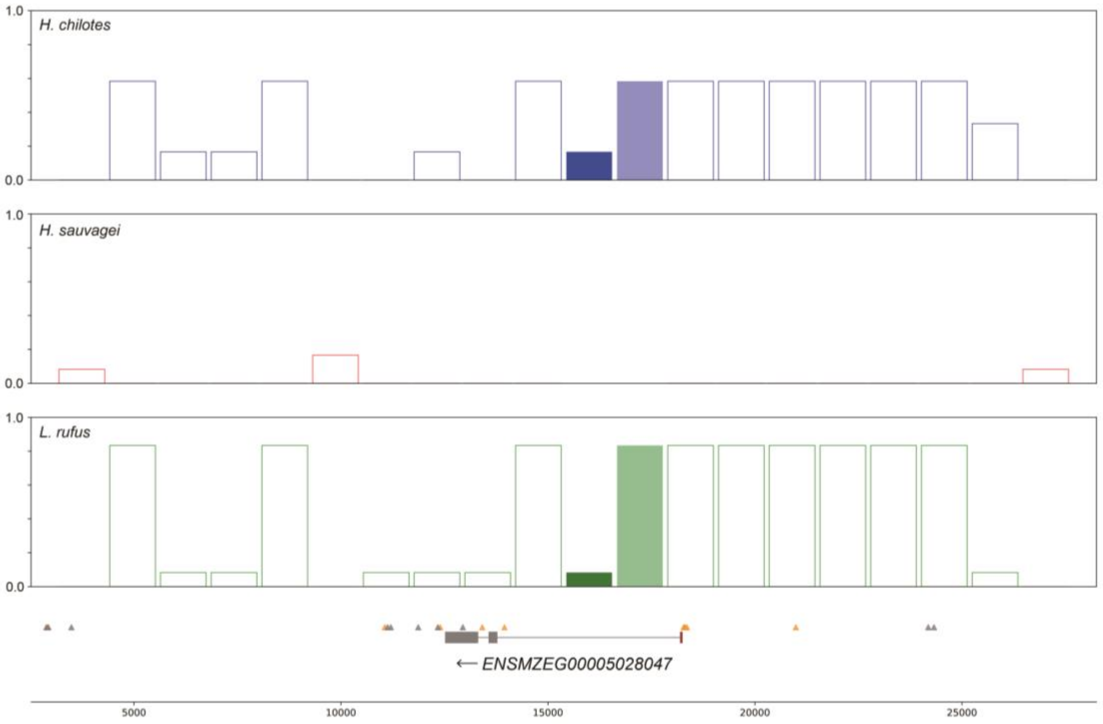


*ENSONIG00000016215* (XP_019208276.1 trace amine-associated receptor 13c-like [Oreochromis niloticus])

*ENSONIG00000016217* (XP_013129440.1 trace amine-associated receptor 13c-like [Oreochromis niloticus])

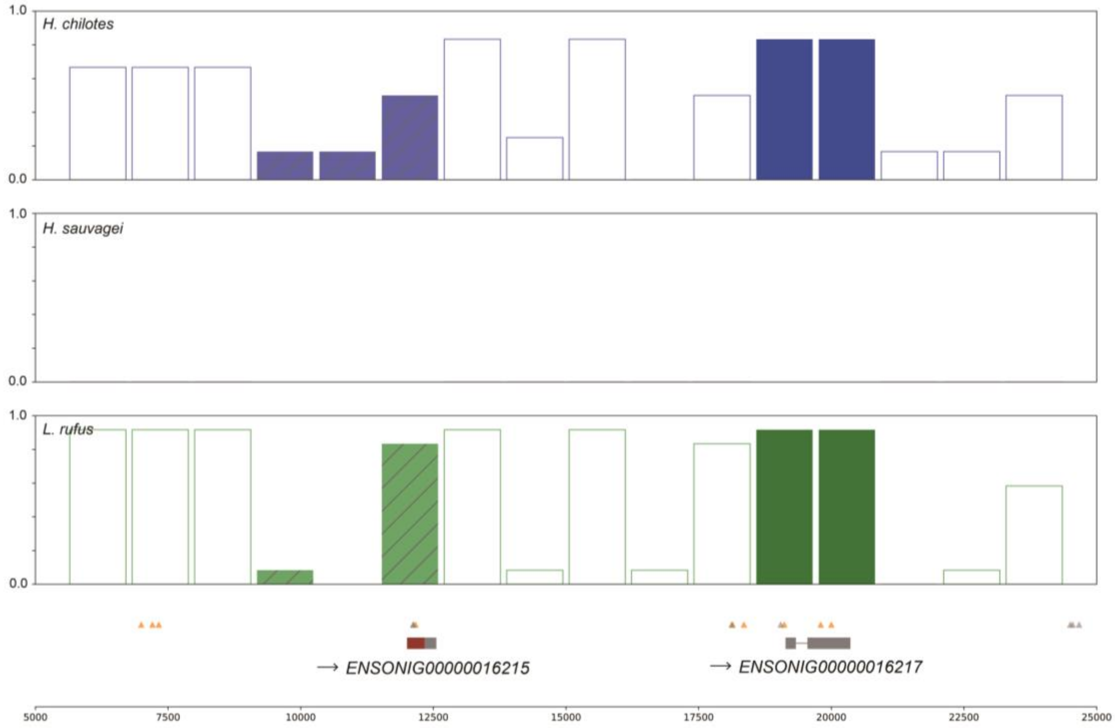

*ENSMZEG00005020407* (XP_023009248.1 trace amine-associated receptor 13c-like [Maylandia zebra])

*ENSMZEG00005020410* (XP_024661068.1 trace amine-associated receptor 13c-like [Maylandia zebra])

*ENSMZEG00005020415* (XP_023009448.2 trace amine-associated receptor 13c-like [Maylandia zebra])

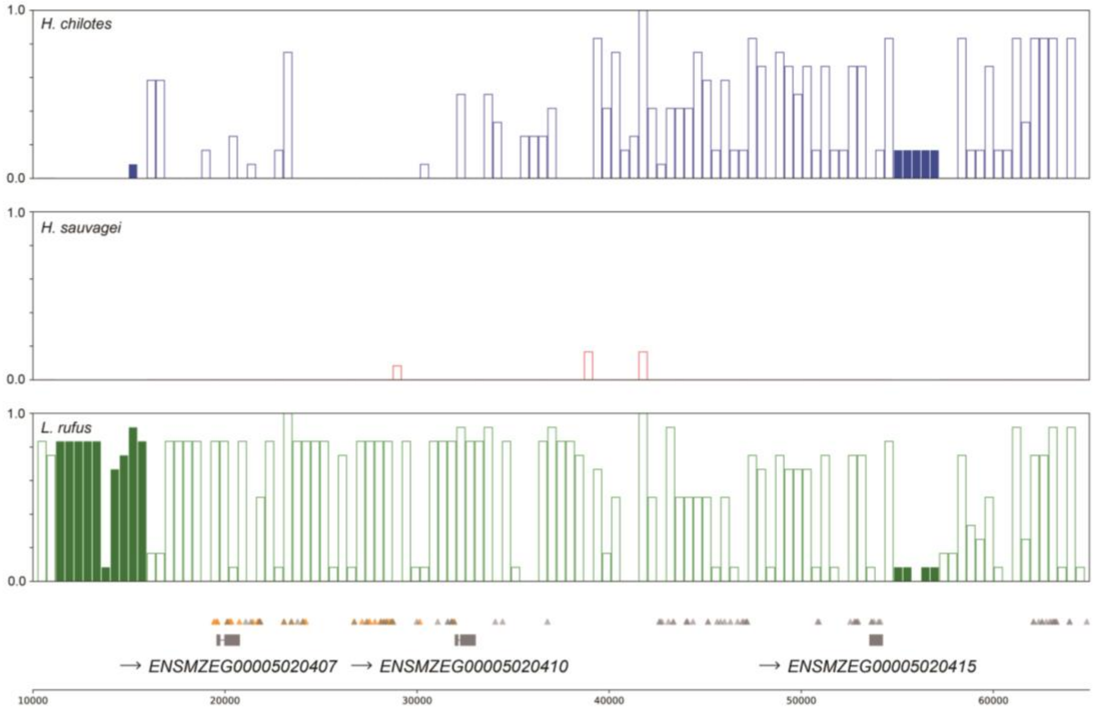


(h) *fga* (XP_013119750.1 fibrinogen alpha chain [Oreochromis niloticus])

*fgb* (XP_003455581.2 fibrinogen beta chain [Oreochromis niloticus])

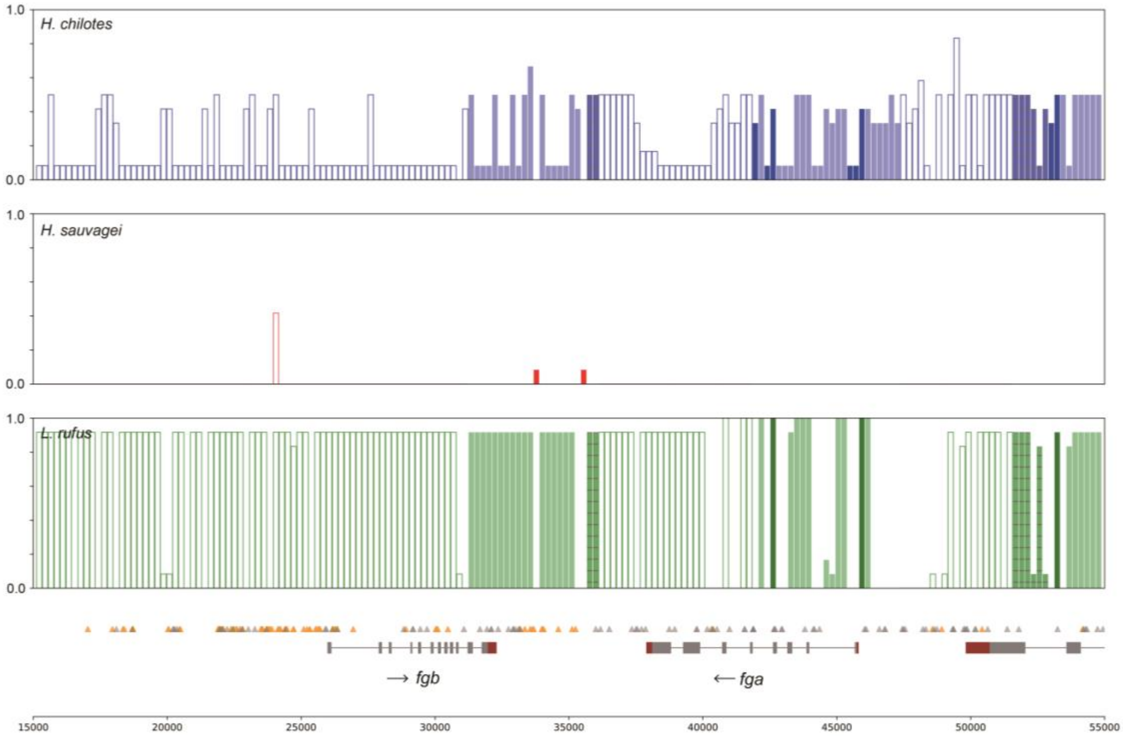

(i) *trpm4*

*ENSONIG00000020074* (XP_019218187.1 transient receptor potential cation channel subfamily M member 4 [Oreochromis niloticus])

*ENSONIG00000020075* (XP_019218186.1 transient receptor potential cation channel subfamily M member 4 [Oreochromis niloticus])

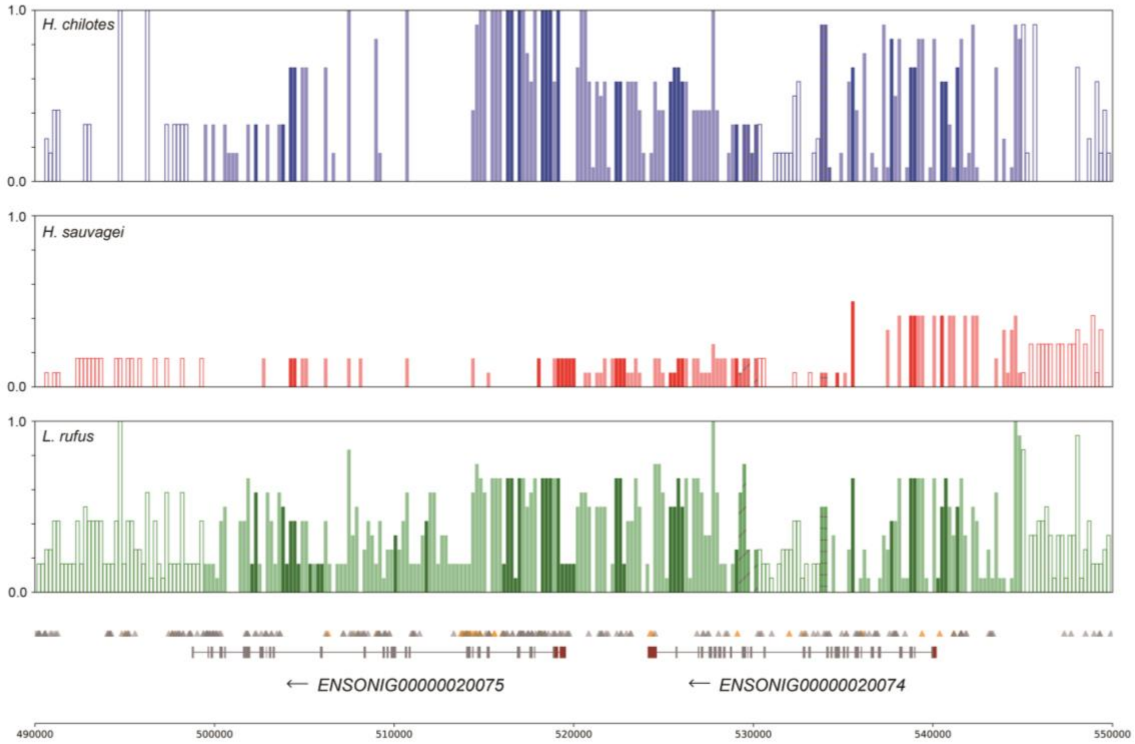

(j) *ENSMZEG00005001611* (XP_024659051.1 intestinal mucin-like protein [Maylandia zebra])

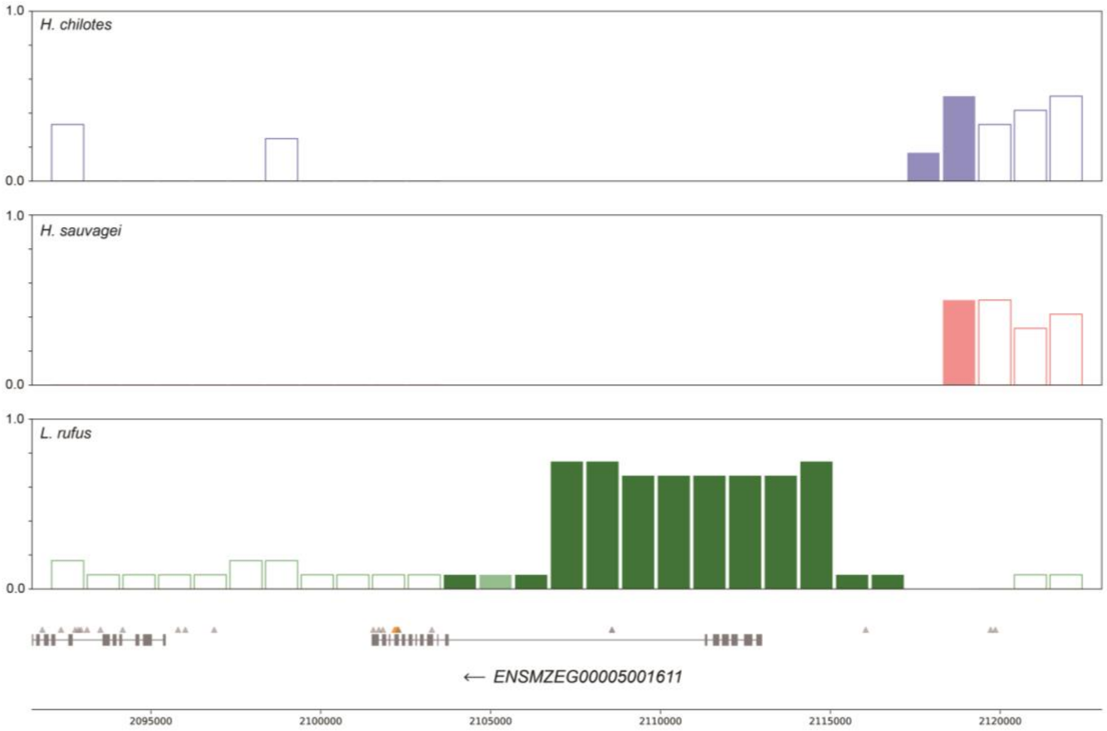


(k) *gtf2f2a* (XP_005466907.1 general transcription factor IIF subunit 2 isoform X1 [Oreochromis niloticus])

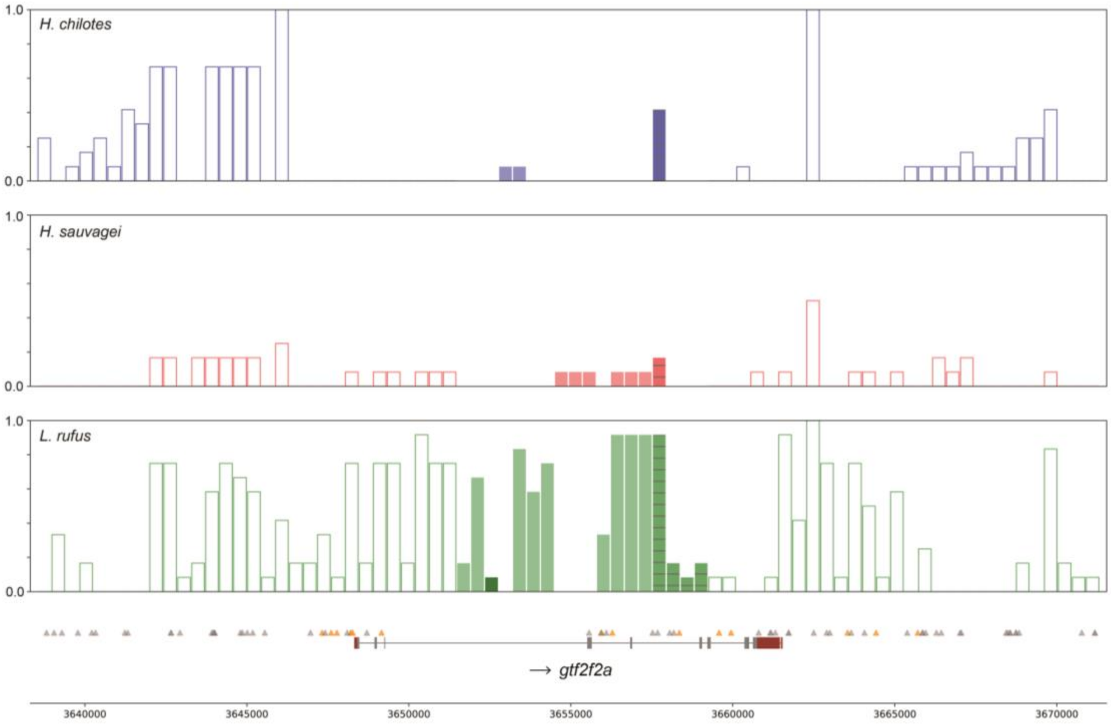

**Fig. S5.** Allele frequency of SNPs on the genomic region of candidate genes on HDRs among three species of Lake Victoria cichlids. Vertical axis shows allele frequencies and horizontal axis shows the relative position of each SNP in the HDRs. Note that the allele frequencies of *H. sauvagei* do not exceed 0.5 because the SNPs were identified against the major alleles of *H. sauvagei*. Vertical bars of a deep shade represent the SNPs in exon regions, and those of a light shade indicate the SNPs in intron regions. Open vertical bars indicate the SNPs in the surrounding regions of two *COL6A6* genes. In the lowest plot, the triangles show the position of SNPs on the scaffold. Particularly, SNPs exhibiting large differences in allele frequencies (>= 0.6) between pairs of species are highlighted by orange based on the patterns of the genomic differentiation. (a) and (f) are *chilotes*-specific genes. (b), (c), (h), three genes of (g), *ENSMZEG00005020407*, *ENSMZEG00005020410* and *ENSMZEG00005020415*, and (k) are *rufus*-specific genes. (d), (e) and (i) are *chilotes-sauvagei* genes. Other genes of (g) are located on *sauvagei*-rufus HDRs. (j) is *chilotes-rufus* genes.

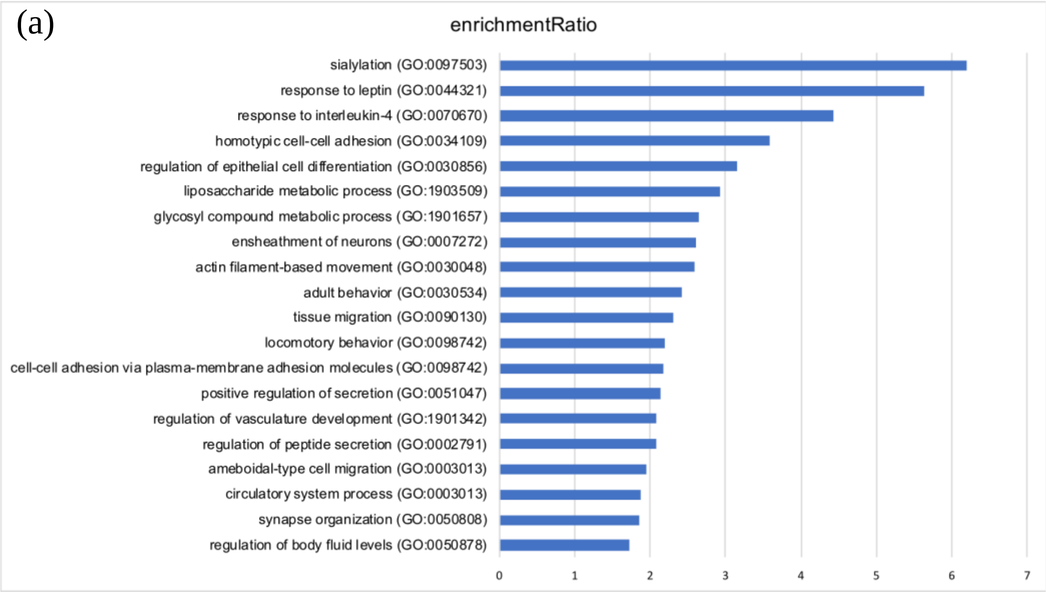


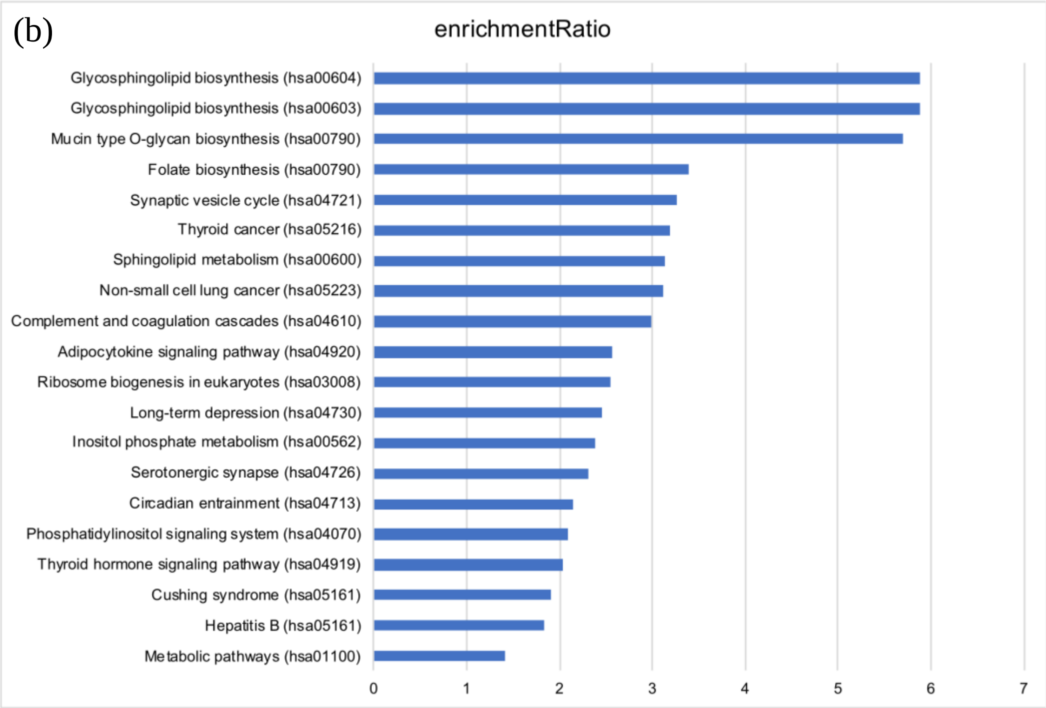


**Fig. S6.** Results of GO enrichment analyses by WebGestalt. Top 20 GO terms obtained under (a) biological process and (b) pathway as functional database are listed.

(a) *ENSMZEG00005012708* (XP_014266774.1 cytochrome P450 2K1 [Maylandia zebra])

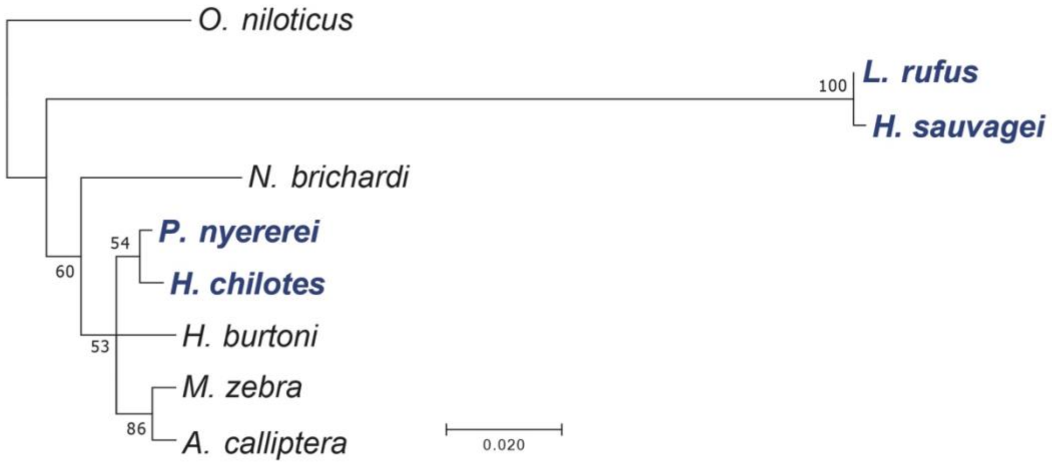


(b) *stat2* (XP_003441521.1 signal transducer and activator of transcription 1-alpha/beta isoform X1 [Oreochromis niloticus])

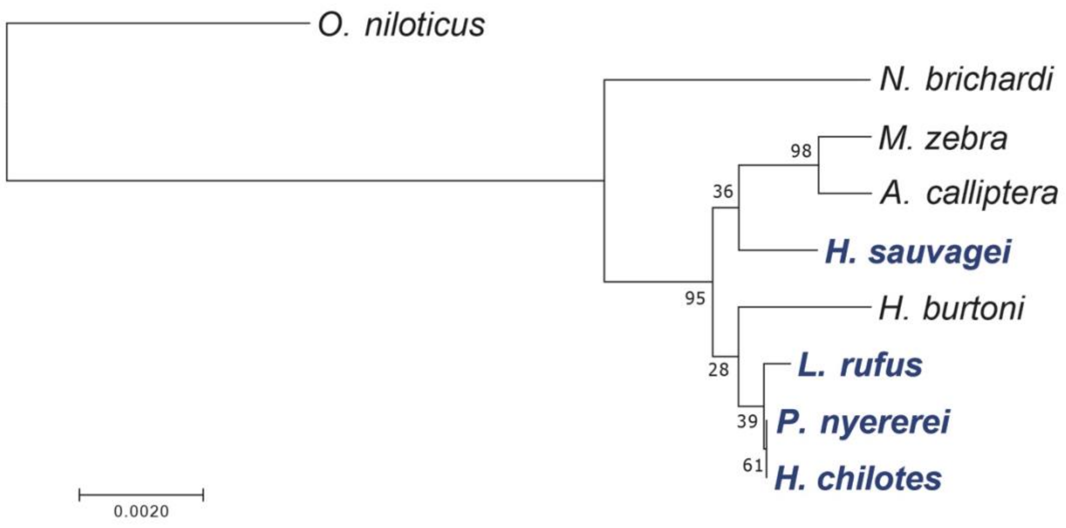

(c) *plppr4b* (XP_005456534.1 phospholipid phosphatase-related protein type 4 [Oreochromis niloticus])

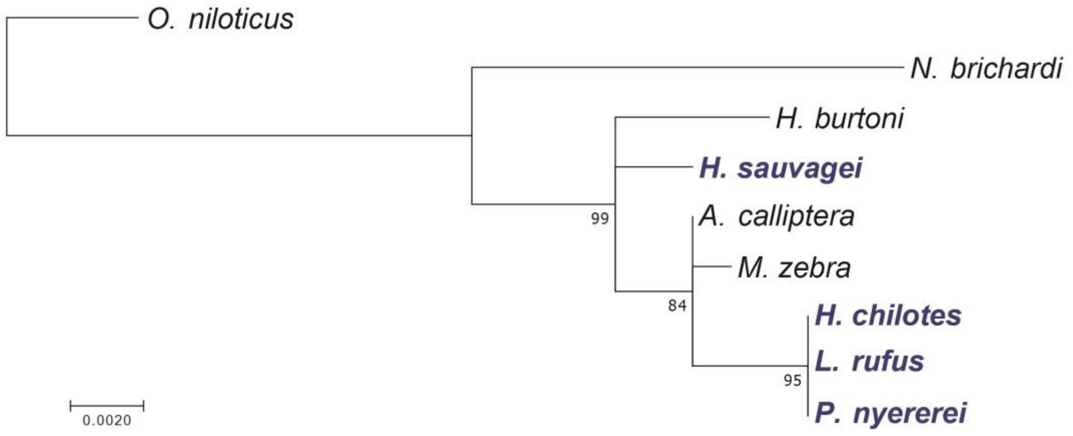


(d) *cdhr5b* (XP_005454335.1 cadherin-related family member 5-like isoform X1 [Oreochromis niloticus])

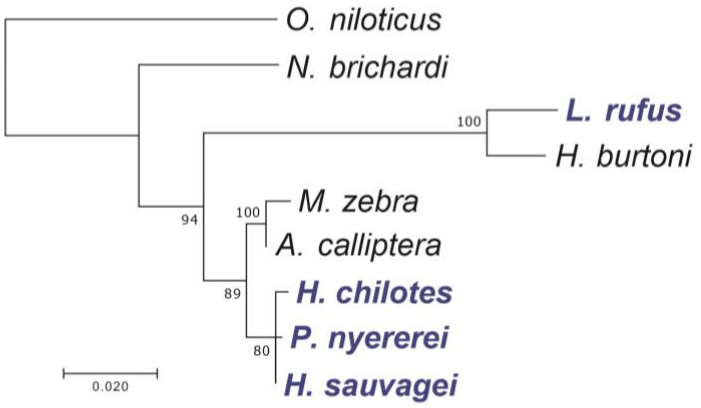

(e) *gtf2f2a* (XP_005466907.1 general transcription factor IIF subunit 2 isoform X1 [Oreochromis niloticus])

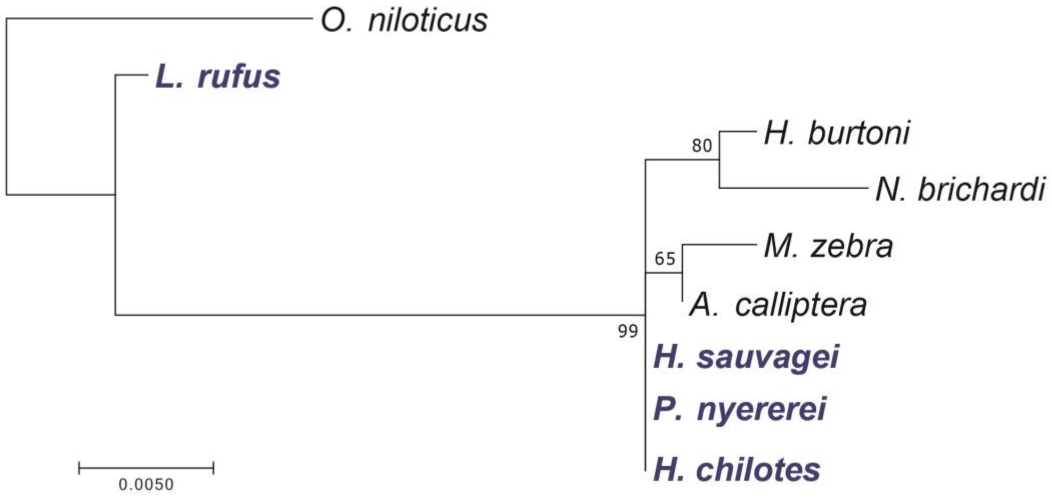


(f) *fga* (XP_013119750.1 fibrinogen alpha chain [Oreochromis niloticus])

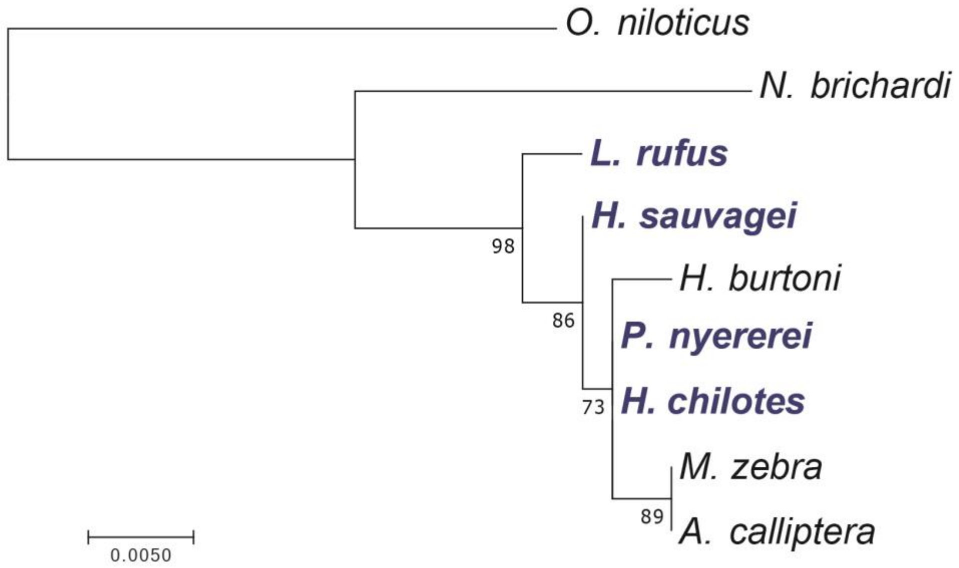

(g) *fgb* (XP_003455581.2 fibrinogen beta chain [Oreochromis niloticus])

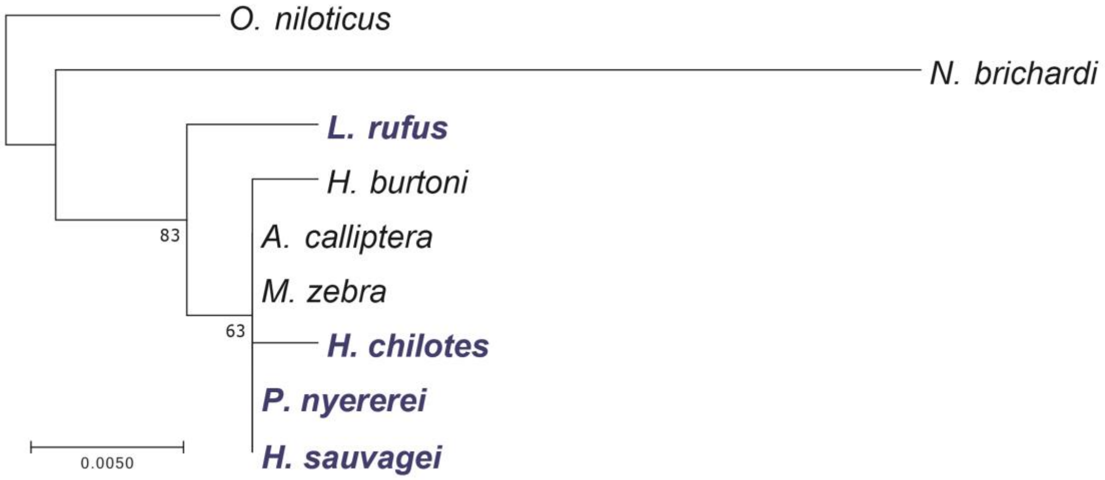


(h) *ctsl* (XP_003453258.1 cathepsin L1 isoform X1 [Oreochromis niloticus])

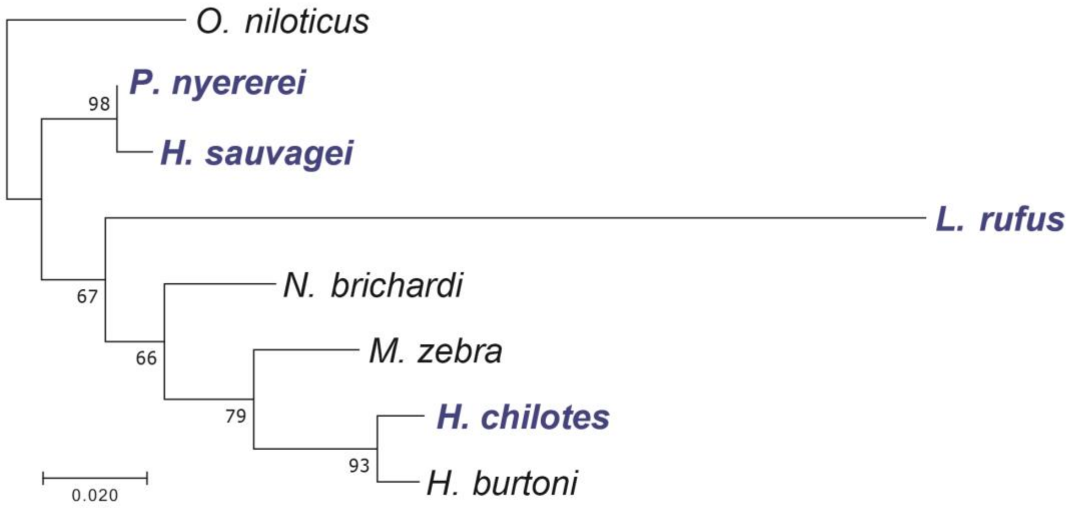

(i) *ENSONIG00000020074* (XP_019218187.1 transient receptor potential cation channel subfamily M member 4 [Oreochromis niloticus])

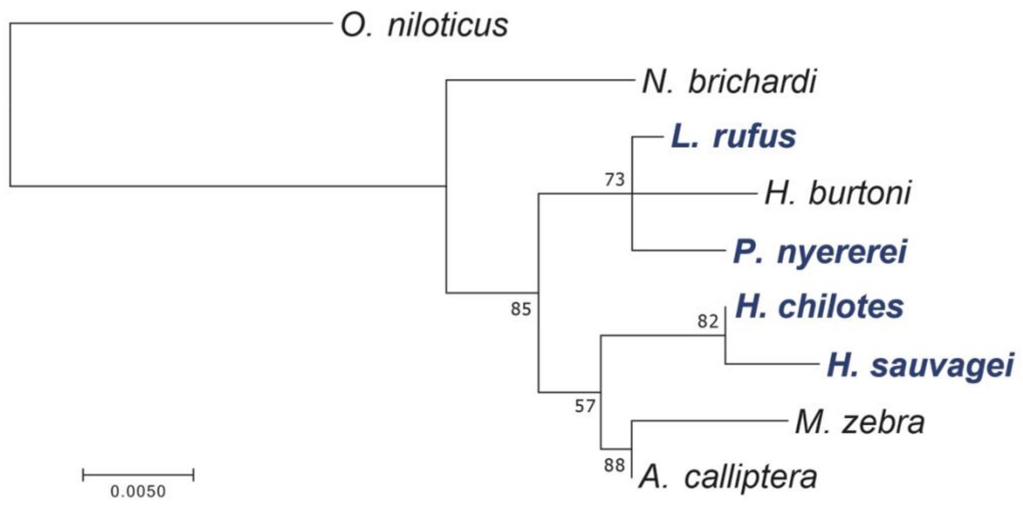


(j) *ENSMZEG00005001611* (XP_024659051.1 intestinal mucin-like protein [Maylandia zebra])

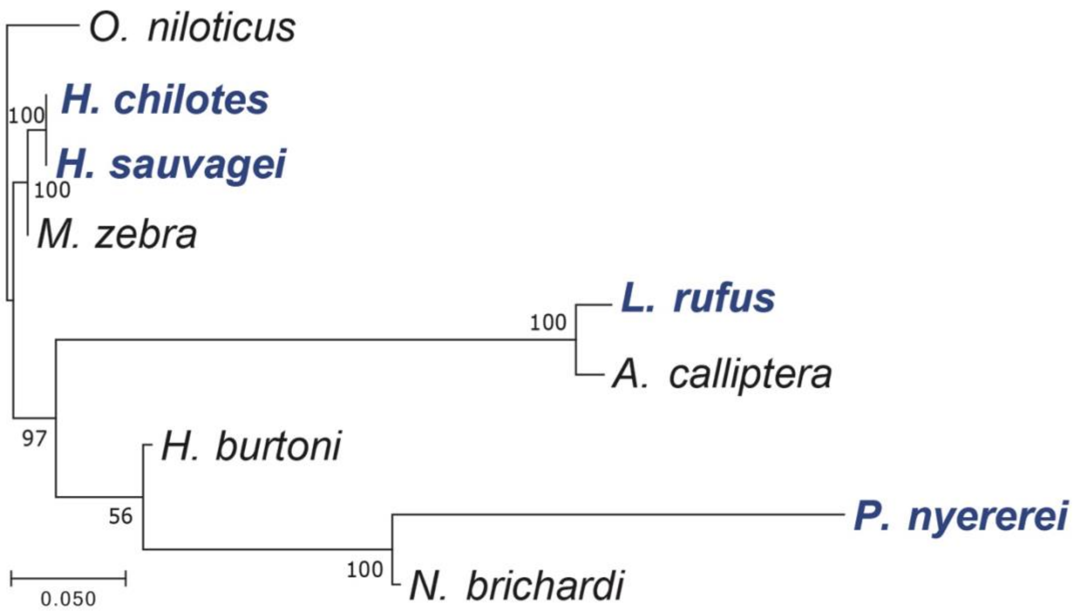

(k) *ENSMZEG00005022254* (XP_025757175.1 membrane cofactor protein isoform X1 [Oreochromis niloticus])

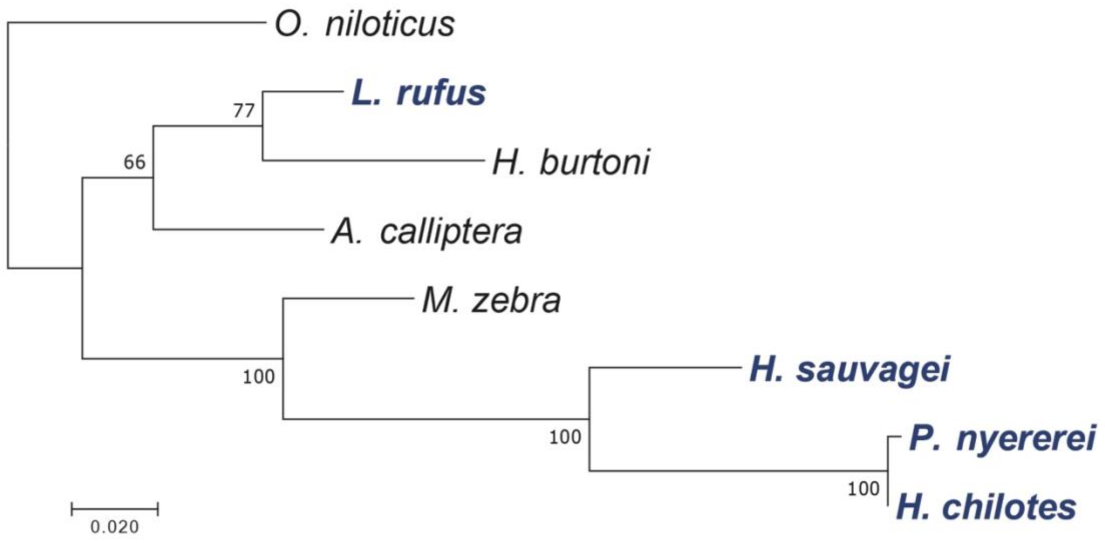


(l) *ENSONIG00000014114* (XP_013119752.1 toll-like receptor 2 [Oreochromis niloticus])

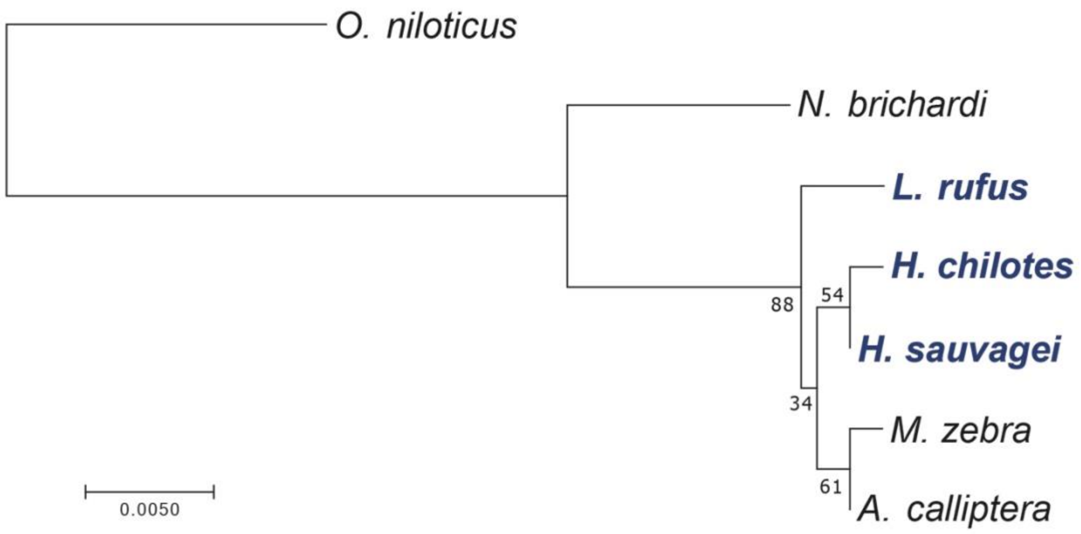

(m) *ENSMZEG00005005638* (XP_014268336.2 TRPM8 channel-associated factor homolog [Maylandia zebra])

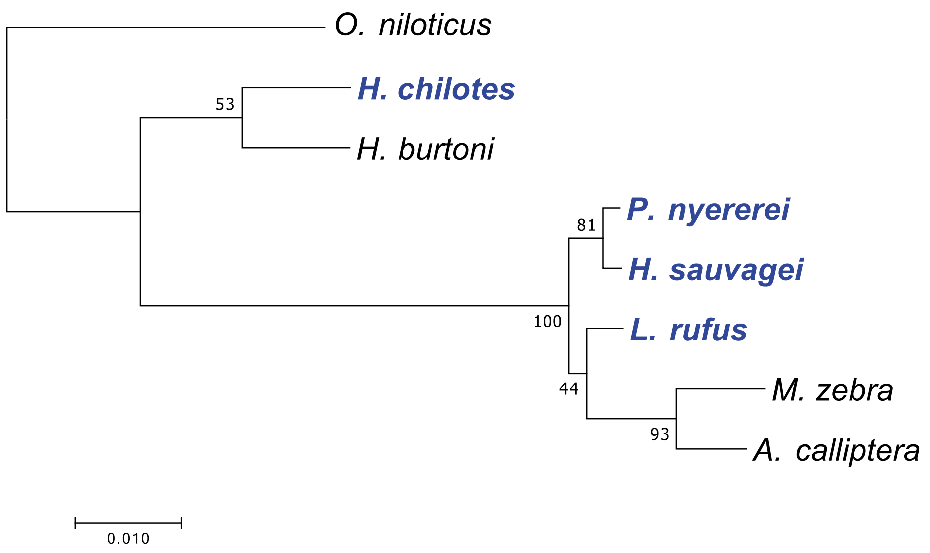


(n) *ENSMZEG00005005677* (XP_024660658.1 TRPM8 channel-associated factor homolog [Maylandia zebra])

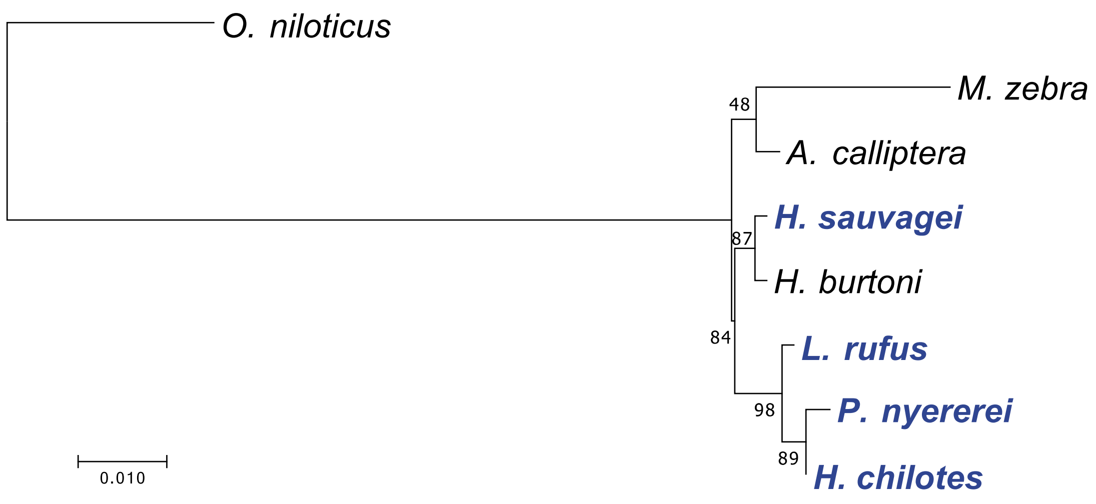

**Fig. S7.** Maximum likelihood estimated phylogenetic trees with 100 bootstrap replicates of candidate genes which are described in Table 1. These may have divergent alleles derived from standing genetic variation. Note that some species were not included in the species tree because their genome sequences were fragmented due to assembly problems.

| Species | Sample | Sampling Point | | | | | | | | | | | | | | | | | | | | | | | | | | | | | | | | | | | |
|---|---|---|---|---|---|---|---|---|---|---|---|---|---|---|---|---|---|---|---|---|---|---|---|---|---|---|---|---|---|---|---|---|---|---|---|---|---|
| *H. chilotes* | HC01 | Nyegezi | C | T | A | A | G | C | A | A | A/G | A | C | A | A | A | T | A | C | C | A | T | G | C | A | A | A | G | T | A | A | T | G | T | A | G | G |
| | HC02 | Chamagati Island | . | . | . | . | . | . | . | . | A/G | . | . | . | . | . | . | . | . | . | . | . | . | . | . | . | . | . | . | . | . | . | . | . | . | . | . |
| | HC03 | Makobe Island | . | . | . | . | . | . | . | . | . | . | . | . | . | . | . | . | . | . | . | . | . | . | . | . | . | . | . | . | . | . | . | . | . | . | . |
| | HC04 | Kilimo Island | C/T | T/C | . | A/G | G/A | C/G | A/G | A/G | A/G | A/T | C/G | A/T | A/G | A/G | T/C | A/T | C/A | . | A/G | T/G | G/A | C/T | A/C | A/T | A/G | G/C | T/G | A/C | A/C | T/A | G/A | T/C | A/G | G/A | G/C |
| | HC05 | Chamagati Island | . | . | . | . | . | . | . | . | A/G | . | . | . | . | . | . | . | . | . | . | . | . | . | . | . | . | . | . | . | . | . | . | . | . | . | . |
| | HC06 | Chamagati Island | . | . | . | . | . | . | . | . | A/G | . | . | . | . | . | . | . | . | . | . | . | . | . | . | . | . | . | . | . | . | . | . | . | . | . | . |
| *H. sauvagei* | HS07 | Bwiru Peninsula | . | T/C | . | A/G | G/A | C/G | . | A/G | . | A/T | C/G | A/T | A/G | A/G | T/C | A/T | C/A | . | A/G | T/G | G/A | C/T | A/C | A/T | A/G | G/C | T/G | A/C | A/C | T/A | G/A | T/C | . | . | . |
| | HS08 | Nyegezi | . | C | G | G | A | G | G | G | . | T | G | T | G | G | C | T | A | . | G | G | A | T | C | T | G | C | G | C | C | A | A | C | . | . | . |
| | HS09 | Chamagati Island | . | . | . | . | . | . | . | . | . | . | . | . | . | . | . | . | . | . | . | . | . | . | . | . | . | . | . | . | . | . | . | . | . | . | . |
| | HS10 | Chamagati Island | . | T/C | A/G | A/G | G/A | C/G | A/G | A/G | . | A/T | C/G | A/T | A/G | A/G | T/C | A/T | C/A | . | A/G | T/G | G/A | C/T | A/C | A/T | A/G | G/C | T/G | A/C | A/C | T/A | G/A | T/C | . | . | . |
| | HS11 | Chamagati Island | . | C | G | G | A | G | G | G | . | T | G | T | G | G | C | T | A | . | G | G | A | T | C | T | G | C | G | C | C | A | A | C | . | . | . |
| | HS12 | Chamagati Island | . | . | . | . | . | . | . | . | . | . | . | . | . | . | . | . | . | . | . | . | . | . | . | . | . | . | . | . | . | . | . | . | . | . | . |
| *L. rufus* | LR13 | Nyaruwambu | . | T/C | G | G | A | G | G | G | . | T | G | T | G | G | C | T | A | C/T | G | G | A | T | C | T | G | C | G | C | C | A | A | C | A/G | G/A | . |
| | LR14 | Nyaruwambu | . | T/C | G | G | A | G | G | G | . | T | G | T | G | G | C | T | A | . | G | G | A | T | C | T | G | C | G | C | C | A | A | C | . | . | . |
| | LR15 | Nyegezi | . | T/C | G | G | A | G | G | G | . | T | G | T | G | G | C | T | A | . | G | G | A | T | C | T | G | C | G | C | C | A | A | C | . | . | . |
| | LR16 | Kilimo Island | C/T | T/C | A/G | G | A | C/G | G | G | . | T | G | T | G | G | C | T | A | . | G | G | G/A | C/T | A/C | A/T | A/G | G/C | T/G | A/C | A/C | T/A | G/A | T/C | A/G | G/A | G/C |
| | LR17 | Kilimo Island | . | . | A/G | A/G | G/A | C/G | A/G | A/G | . | T | G | A/T | A/G | A/G | T/C | A/T | C/A | . | A/G | T/G | G/A | C/T | A/C | A/T | A/G | G/C | T/G | A/C | A/C | T/A | G/A | T/C | . | . | . |
| | LR18 | Nyaruwambu | C/T | C | G | G | A | C/G | G | G | . | T | G | T | G | G | C | T | A | . | G | G | A | T | C | T | G | C | G | C | C | A | A | C | A/G | G/A | G/C |

**Fig. S8.** Allelic patterns on coding regions of *COL6A6*_a, which is located on *chilotes*-*rufus* HDRs. Coding regions were predicted by GeneWise and were executed multiple alignment in MEGA7. Only polymorphic sites are shown, especially, non-synonymous mutations are highlighted by yellow.

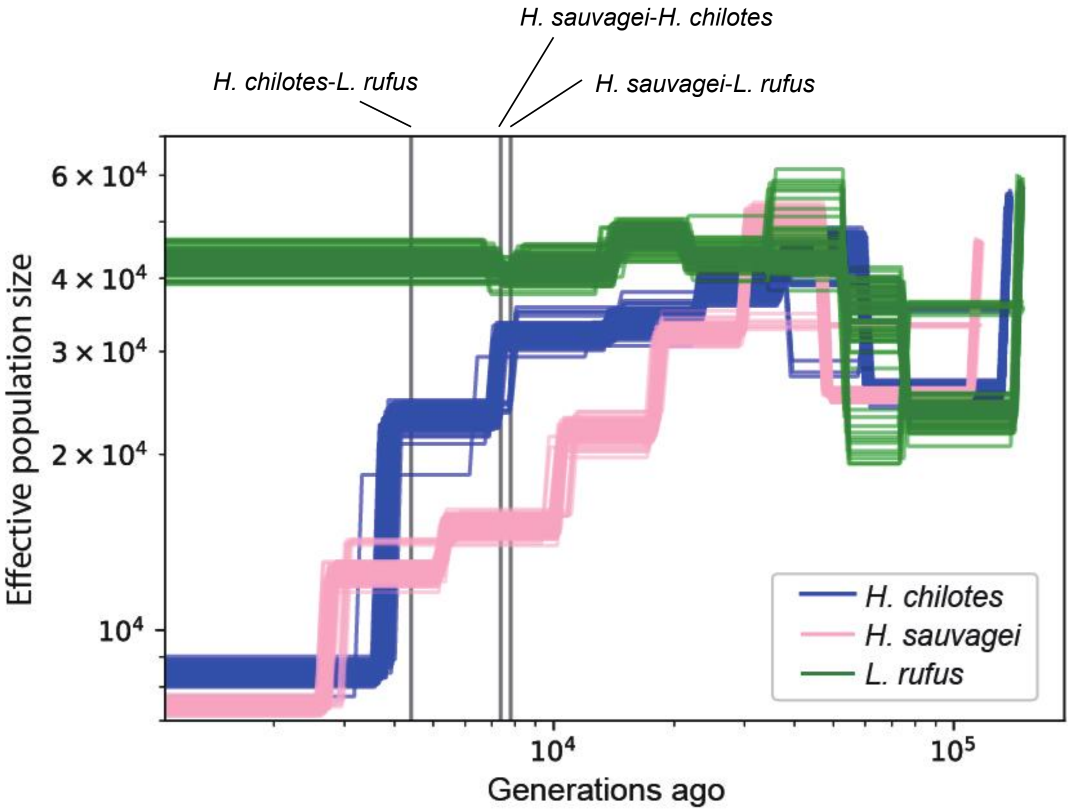


**Fig. S9.** Patterns of changes in population size of each species over generation estimated by *smc++* analyses with 100 bootstrap replicates. All results are estimated by using 12 unphased haplotypes (six individuals of each species) data comprised from SNPs on the same scaffolds as *msmc2* analyses. Blue lines show the estimated patterns of changes in population size of *H. chilotes*, pink lines show those of *H. sauvagei* and green lines show those of *L. rufus*. The average estimated timings of speciation between pair of species are shown by gray vertical lines in figure 2c. Mutation rate is used $3.5\times10^{-9}$ mutations per base pair per generation (Malinsky et al. 2018).

| Species | Sample | Sampling Point | LWS 26 | 62 | 131 | 137 | 168 | 177 | 179 | 191 | 216 | 222 | 226 | 227 | 230 | 275 | 282 | 317 | Alleles |
|---|---|---|---|---|---|---|---|---|---|---|---|---|---|---|---|---|---|---|---|
| *H. chilotes* | HC01 | Nyegezi | A | V | S | A | A | A | V | Y | Y | L | I | F | A | C | T | I | H |
| | HC02 | Chamagati Island | . | . | . | . | . | . | . | . | . | . | . | . | . | . | . | . | H |
| | HC03 | Makobe Island | . | . | . | . | . | . | . | . | F | . | . | . | T | I | . | . | P |
| | HC04 | Kilimo Island | . | . | . | . | . | . | . | . | . | . | . | . | . | . | . | . | H |
| | HC05 | Chamagati Island | . | . | . | . | . | . | . | . | . | . | . | . | . | . | . | . | H |
| | HC06 | Chamagati Island | . | . | . | . | . | . | . | . | . | . | . | . | . | . | . | . | H |
| *H. sauvagei* | HS07 | Bwiru Peninsula | . | . | . | . | . | . | . | . | . | . | . | . | . | . | . | . | H |
| | HS08 | Nyegezi | . | . | . | . | . | . | . | . | . | . | . | . | . | . | . | . | H |
| | HS09 | Chamagati Island | . | . | . | . | . | . | . | . | . | . | . | . | . | . | . | . | H |
| | HS10 | Chamagati Island | . | . | . | . | . | . | . | . | . | . | . | . | . | . | . | . | H |
| | HS11 | Chamagati Island | . | . | . | . | . | . | . | . | . | . | . | . | . | . | . | . | H |
| | HS12 | Chamagati Island | . | . | . | . | . | . | . | . | . | . | . | . | . | . | . | . | H |
| *L. rufus* | LR13 | Nyaruwambu | V | I | . | G | G | S | F | F | . | I | . | I | S | . | . | V | new |
| | LR14 | Nyaruwambu | V | I | . | G | G | S | F | F | . | I | . | I | S | . | . | V | new |
| | LR15 | Nyegezi | V | I | . | G | G | S | F | F | . | I | . | I | S | . | . | V | new |
| | LR16 | Kilimo Island | V | I | . | G | G | S | F | F | . | I | . | I | S | . | . | V | new |
| | LR17 | Kilimo Island | V | I | . | G | G | S | F | F | . | I | . | I | S | . | . | V | new |
| | LR18 | Nyaruwambu | V | V/I | . | A/G | A/G | A/S | V/F | Y/F | . | L/I | . | F/I | S/T | C/I | . | I/V | new |

| Species | Sample | Sampling Point | RH1 33 | 42 | 95 | 104 | 158 | 213 | 217 | 263 | 269 | 298 | Alleles |
|---|---|---|---|---|---|---|---|---|---|---|---|---|---|
| *H. chilotes* | HC01 | Nyegezi | S | C | V | V/I | G | L | T | I | A | A | r104V / r104I |
| | HC02 | Chamagati Island | . | . | . | . | . | . | . | . | . | . | r104V |
| | HC03 | Makobe Island | . | . | . | I | . | . | . | . | . | . | r104I |
| | HC04 | Kilimo Island | . | . | . | V/I | . | . | . | . | . | . | r104V / r104I |
| | HC05 | Chamagati Island | . | . | . | V/I | . | . | . | . | . | . | r104V / r104I |
| | HC06 | Chamagati Island | . | . | . | V/I | . | . | . | . | . | . | r104V / r104I |
| *H. sauvagei* | HS07 | Bwiru Peninsula | . | . | . | I | . | . | . | . | . | . | r104I |
| | HS08 | Nyegezi | . | . | . | I | . | . | . | . | . | . | r104I |
| | HS09 | Chamagati Island | . | . | . | . | . | . | . | . | . | . | r104V |
| | HS10 | Chamagati Island | . | . | . | I | . | . | . | . | . | . | r104I |
| | HS11 | Chamagati Island | . | . | . | V/I | . | . | . | . | . | . | r104V / r104I |
| | HS12 | Chamagati Island | . | . | . | I | . | . | . | . | . | . | r104I |
| *L. rufus* | LR13 | Nyaruwambu | N | . | . | I | . | L/S | . | . | . | . | new |
| | LR14 | Nyaruwambu | N | . | . | I | . | S | . | . | . | . | new |
| | LR15 | Nyegezi | N | . | . | I | . | S | . | . | . | . | new |
| | LR16 | Kilimo Island | N | . | . | I | . | S | . | . | . | . | new |
| | LR17 | Kilimo Island | N | . | . | I | . | S | . | . | . | . | new |
| | LR18 | Nyaruwambu | N | . | . | I | . | S | . | . | . | . | new |

**Fig. S10.** Allele groups of *LWS* and *RH1*. The allele types were identified based on the results of Terai et al. (2017) and the alleles with 'new' indicate newly divergent alleles which were not classified to the known allele groups of Lake Victoria cichlids.

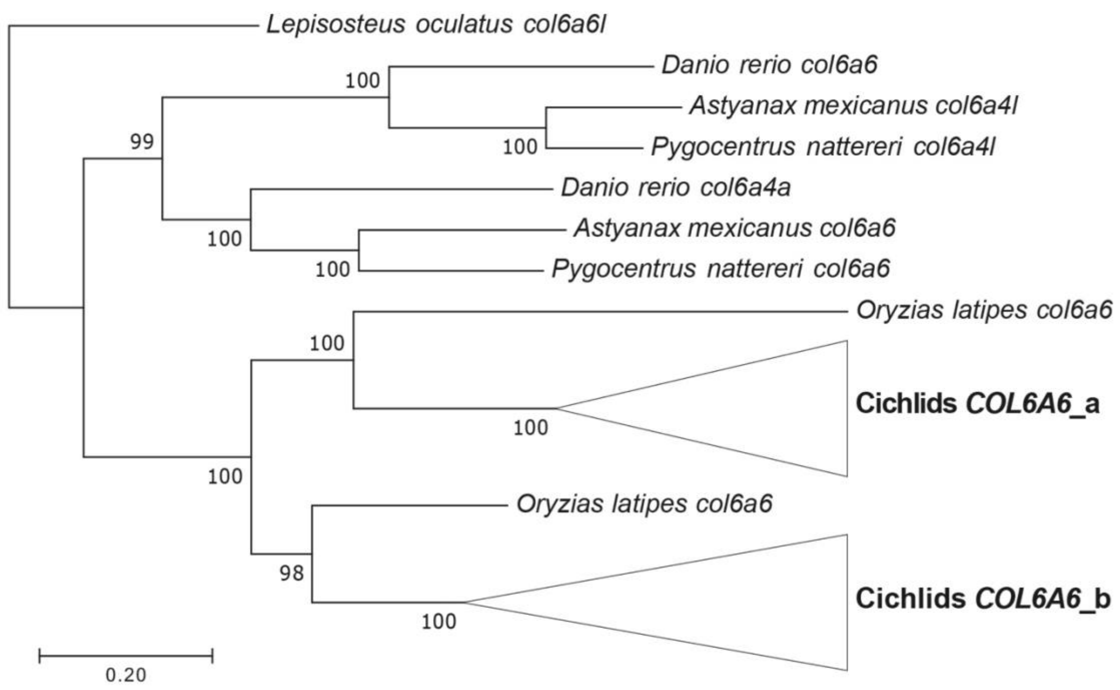


**Fig. S11.** Maximum likelihood estimated phylogenetic tree with 100 bootstrap replicates of two *COL6A6* genes. We used amino acid sequences from *Lepisosteus oculatus* (outgroup species), *Danio rerio*, *Astyanax mexicanus*, *Pygocentrus nattereri* and *Oryzias latipes*, beside East African cichlids.
